# Variance-aware weight quantization of multi-level resistive switching devices based on Pt/LaAlO_3_/SrTiO_3_ heterostructures

**DOI:** 10.1038/s41598-022-13121-4

**Published:** 2022-05-31

**Authors:** Sunwoo Lee, Jaeyoung Jeon, Kitae Eom, Chaehwa Jeong, Yongsoo Yang, Ji-Yong Park, Chang-Beom Eom, Hyungwoo Lee

**Affiliations:** 1 Department of Electrical and Computer Engineering, University of Southern California, Los Angeles, CA 90007 USA; 2 Department of Physics, Ajou University, Suwon, 16499 Republic of Korea; 3 Department of Energy Systems Research, Ajou University, Suwon, 16499 Republic of Korea; 4 Department of Materials Science and Engineering, University of Wisconsin-Madison, Madison, WI 53706 USA; 5 Department of Physics, Korea Advanced Institute of Science and Technology (KAIST), Daejeon, 34141 Republic of Korea

**Keywords:** Materials science, Nanoscience and technology

## Abstract

Resistive switching devices have been regarded as a promising candidate of multi-bit memristors for synaptic applications. The key functionality of the memristors is to realize multiple non-volatile conductance states with high precision. However, the variation of device conductance inevitably causes the state-overlap issue, limiting the number of available states. The insufficient number of states and the resultant inaccurate weight quantization are bottlenecks in developing practical memristors. Herein, we demonstrate a resistive switching device based on Pt/LaAlO_3_/SrTiO_3_ (Pt/LAO/STO) heterostructures, which is suitable for multi-level memristive applications. By redistributing the surface oxygen vacancies, we precisely control the tunneling of two-dimensional electron gas (2DEG) through the ultrathin LAO barrier, achieving multiple and tunable conductance states (over 27) in a non-volatile way. To further improve the multi-level switching performance, we propose a variance-aware weight quantization (VAQ) method. Our simulation studies verify that the VAQ effectively reduces the state-overlap issue of the resistive switching device. We also find that the VAQ states can better represent the normal-like data distribution and, thus, significantly improve the computing accuracy of the device. Our results provide valuable insight into developing high-precision multi-bit memristors based on complex oxide heterostructures for neuromorphic applications.

## Introduction

Resistive switching devices are one of the leading candidates for memristors for synaptic applications^1–4^. In recent years, research effort has been focused on their capability of multi-level non-volatile switching, aiming for a high-level in-memory computation^5–11^. The ultimate goal of the memristors is an analog operation with a nearly infinite number of conductance states, imitating the analog operation of biological synapses. Considering the mechanism of resistive switching, in principle, most of conventional memristors should be able to stabilize a tremendous number of conductance states with a separation of a conductance quantum *G*_0_ = 2*e*^2^/*h*, where *e* and *h* represents the unit charge and Plank’s constant, respectively^12–14^. However, because the uncontrolled resistive switching mechanisms and the defect-induced charge trapping phenomena inevitably cause the random fluctuation of output current, the weight values of the memristors always show non-negligible variation^14,15^. Thus, the conductance states of the memristors are often quantized so that the output signal with variation can be rounded to the nearest state^16^. In this way, the state-overlap issue can be circumvented, but the number of available states (i.e., the number of the representable weight values) severely decreases. At this stage, increasing the on/off conductance ratio of the devices is required to maximize the number of conductance states. The minimization of current fluctuation is also essential to fully take advantage of the limited conductance range.

Additionally, it is worth considering how to define the conductance states of the memristors. In most of the previous research, the multiple conductance states of memristors are defined by uniformly dividing the available conductance range. That is probably because the uniform states can be simply programmed by linearly-incremental forming voltages. However, based on the knowledge of neural network training, the actual weight values as well as intermediate data during training, such as activations, commonly have a normal-like distribution^17,18^. This implies that the small-weight-regime contributes to the result of the training more dominantly than the high-weight-regime. Therefore, the conventional uniform configuration of the conductance states may not be optimal for achieving high accuracy of the memristor-based computations. The non-uniform conductance states, configured considering the data characteristics, will better represent the distribution of the weights and the activations. Therefore, to improve the fundamental performance of memristors, we focus on two issues: (1) to build an advanced resistive switching device that can realize a larger number of conductance states and (2) to appropriately define its conductance states for higher quantization accuracy.

Herein, we demonstrate a resistive switching device based on Pt/LaAlO_3_/SrTiO_3_ (Pt/LAO/STO) heterostructure, which is suitable for multi-level memristive applications. By redistributing the surface oxygen vacancies, which create defect states in the band gap of LAO, we precisely control the tunneling of two-dimensional electron gas (2DEG) through the ultrathin LAO barrier in a non-volatile way. The 2DEG-based memristive device, namely the 2DEG memristor, achieves multiple conductance states (in excess of 27 states) with high reliability. To further improve the multi-level switching performance of the device, we propose the variance-aware weight quantization (VAQ) method. Our simulation studies verify that the VAQ can effectively reduce the state-overlap issue of the 2DEG memristors. Furthermore, we find that the VAQ states can better represent the normal-like data distribution and, hence, provides greater accuracy in image classification processes. These results will offer a significant step toward developing practical multi-bit memristors based on complex oxide heterostructures for neuromorphic applications.

## Results and discussion

### Resistive switching devices based on Pt/LAO/STO heterostructures

**Figure 1:**
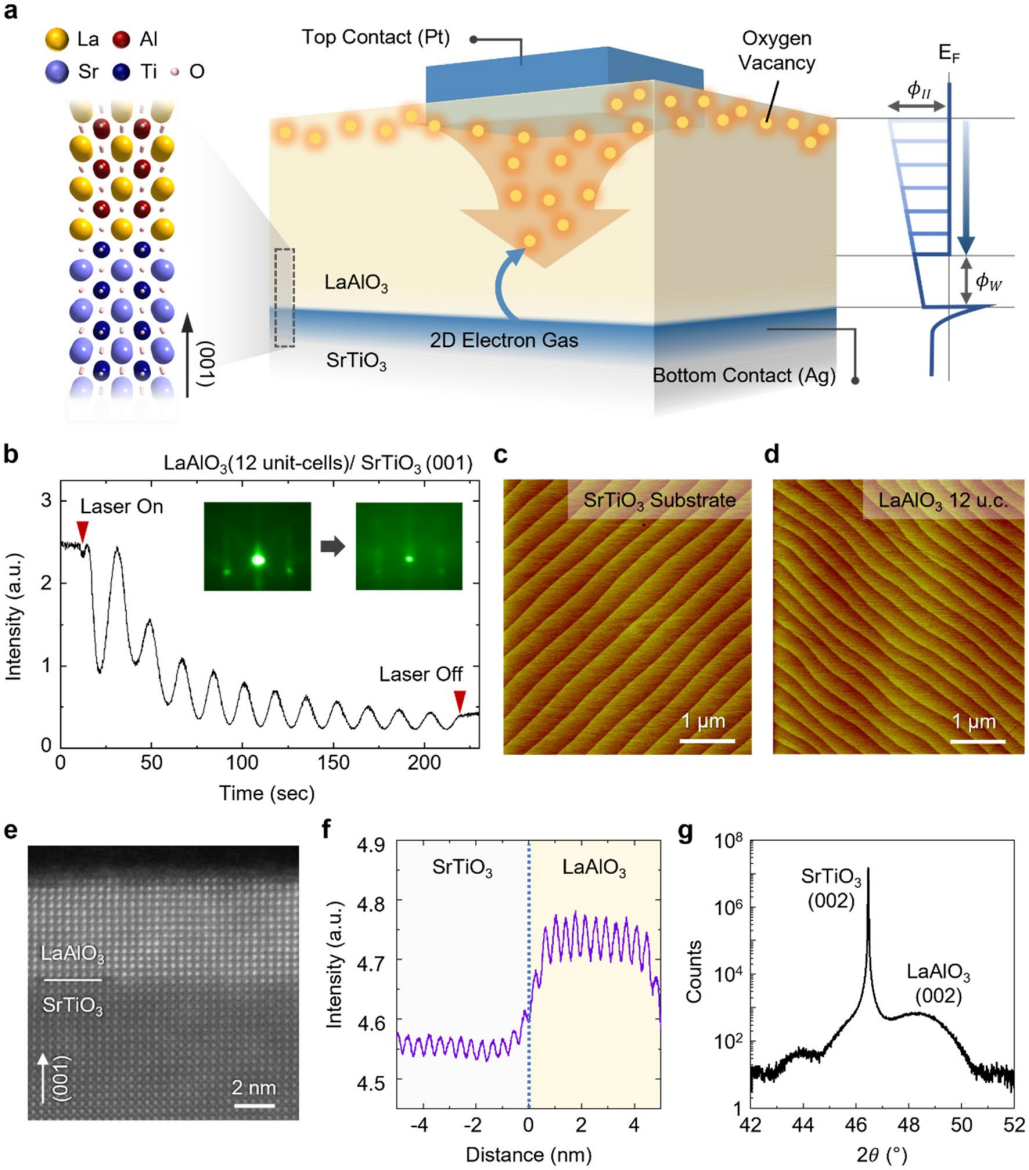
Resistive switching device based on a Pt/LAO/STO heterostructure. (**a**) Schematic depicting the mechanism for the resistive switching in the oxide heterostructure. The spatial distribution of oxygen vacancies determines the tunneling probability of the 2DEG between the LAO/STO interface and the top Pt electrode. (**b**) Thickness-dependent evolution of the in situ RHEED intensity oscillation during the PLD deposition of LAO thin films. The insets show the RHEED patterns before and after the film growth. (**c**) AFM topography image measured on the surface of a thermally-treated STO (001) substrate. (**d**) AFM topography image measured on the surface of an as-grown LAO thin film. (**e**) HAADF-STEM image of the LAO/STO heterostructure. (**f**) Intensity of a line profile along (001) obtained from the STEM image. (**g**) XRD *θ*–2*θ* scan of the LAO/STO heterostructure.

The LAO/STO heterointerfaces have emerged as a new playground for exploring emergent electronic properties. The polarity discontinuity at the LaO^+^/TiO_2_^0^ heterointerface generates an electric field pointing away from the bottom interface to the top surface in the LAO/STO heterostructure^19,20^. The built-in field is necessarily compensated by the formation of 2DEG at the bottom LAO/STO interface^21^. The oxide interface with the 2DEG was found to be highly conducting. Moreover, the 2DEG has shown many interesting physical properties distinct from conventional semiconductor heterostructures^22,23^, and thus offers possibilities for device applications^24–26^. We design a resistive switching device based on the 2DEG in the LAO/STO heterostructures. Figure 1a shows the vertical device configuration of a Pt/LAO/STO heterostructure. The highly-conducting 2DEG at the LAO/STO interface serves as a reliable bottom electrode in this device structure. When a positive bias voltage is applied to the top Pt electrode the 2DEG tunnels through the insulating LAO layer, resulting in a vertical current. We employ oxygen vacancy point defects to modulate the tunneling conductance. Since the oxygen vacancies in the LAO form intermediate energy levels within the bandgap^27^, they can serve as hopping sites for electrons. Thus, the distribution of the oxygen vacancies in LAO determines the effective tunneling probability of the 2DEG across the LAO barrier. Notably, an as-grown LAO thin film has most of its oxygen vacancies at the top surface due to the internal built-in field^20,28^. By redistributing the surface oxygen vacancies, we can form a conducting region, so-called the conducting filament, near the Pt/LAO interface and control the effective conductance across the Pt/LAO/STO junction (see more details in Fig. S1, Supporting Information). Note that, unlike the conventional metal/oxide/metal structures, the surface oxygen vacancies are formed as a counterpart of the buried 2DEG in the Pt/LAO/STO heterostructure^19^. The internal electric field originated from the polarity discontinuity at the LAO/STO interface determines how many electrons are to be accumulated at the bottom interface and similarly how many oxygen vacancies are to be formed at the top surface (Fig. S2, Supporting Information). Thus, by controlling the thickness of the LAO and thereby the internal electric field, we can reproducibly control the densities of both 2DEG and surface oxygen vacancies. This makes the 2DEG heterostructures unique and suitable for resistive switching device applications.

To build the 2DEG memristor, we synthesized a LAO thin film on a TiO_2_-terminated (001) STO substrate by pulsed laser deposition (PLD) with in situ monitoring of reflection high-energy electron diffraction (RHEED). Figure 1b shows the oscillation and the patterns of RHEED, indicating the layer-by-layer growth of the single-crystalline LAO thin film. After the growth of the film, the LAO/STO heterostructure was slowly cooled down to room temperature without oxygen gas injection or post-annealing, so that the oxygen vacancies are not fully removed. The Pt electrodes were subsequently fabricated on the top surface of the LAO thin film through a conventional lift-off process. A commercial Ag paste is used for the bottom contact. We confirmed that the Ag contact does not involve the resistive switching mechanism in the Pt/LAO/STO heterostructures (Sect. 3, Supporting Information). Further details of the sample fabrication are found in the “Methods” section.

Figure 1c,d show the atomic force microscopy (AFM) images measured on the surface of a thermally-treated STO (001) substrate and an as-grown LAO thin film, respectively. The surface of the as-grown LAO film is atomically flat and smooth, indicating the high quality of the film. The step-and-terrace structure on the LAO surface, which is almost identical to that on the STO substrate, implies that the layer-by-layer growth mode is well preserved throughout the deposition process. The high-angle annular dark field scanning transmission electron microscopy (HAADF-STEM) image taken from the same sample (Fig. 1e) also indicates the high quality of the LAO/STO heterostructure. The line profile along (001) from the STEM image (Fig. 1f) confirms that the thickness of the LAO thin film is exactly 12 unit-cells, as we designed, and the atomic intermixing at the interface is minimal. Figure 1g shows the out-of-plane *θ*–2*θ* X-ray diffraction (XRD) pattern around (002) STO peak. Only a single peak representing the (002) reflection of the LAO is found, ensuring the epitaxial nature of the single-crystalline LAO thin film. All of these structural analyses confirm the high crystallinity and the well-defined heterointerface of the LAO/STO heterostructure, regardless of its oxygen deficiency.

**Figure 2:**
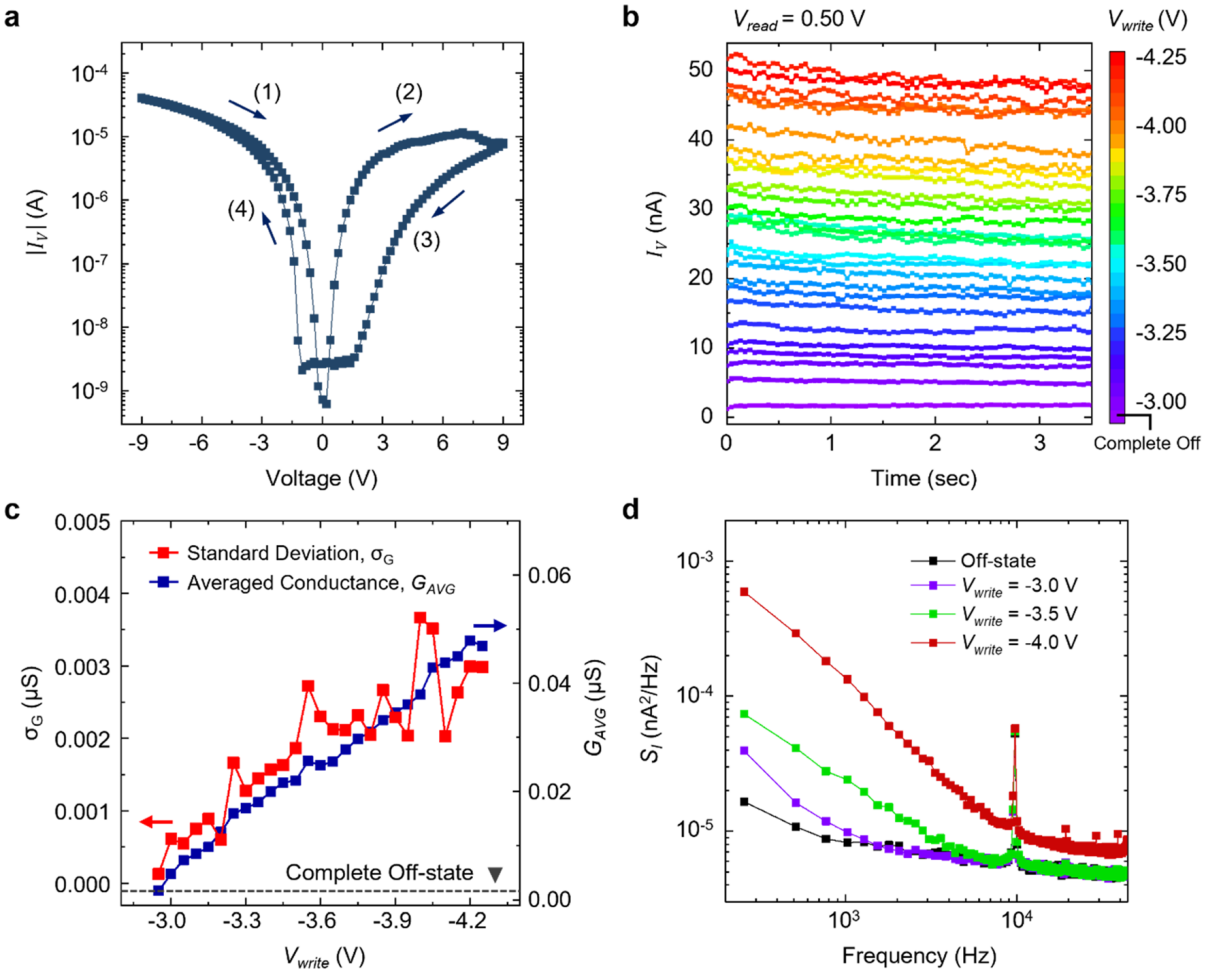
Multi-level switching behavior of the 2DEG memristor. (**a**) *I–V* characteristics of the 2DEG memristor. The arrows represent the directions of the voltage sweep. Note that the initially applied negative voltage switches the device to the on-state (step (1)), while the subsequent positive voltage switches the device back to the off-state (step (2)). (**b**) Sequentially programmed conductance states, showing the representative 27 states. (**c**) The standard deviation (red squares) and the averaged conductance values (blue squares) at all individual conductance states. (**d**) Current power spectral density of output currents at the representative 3 different states and the off-state.

We examined the electrical switching characteristics of the Pt/LAO/STO heterostructure. Figure 2a shows the representative *I–V* curve of the device. As indicated by the pinched hysteresis loop, the device exhibits a bipolar resistance switching behavior. The *I–V* characteristics show that the positive voltage results in the off-switching (i.e., decreasing the tunneling conductance), while the negative voltage results in the on-switching. This switching polarity supports our hypothetical resistive switching mechanism, which is based on the surface oxygen vacancies. The initial conductance of pristine Pt/LAO/STO heterostructures is confirmed to be quite low (Fig. S5, Supporting Information). When a negative voltage is applied to the bottom 2DEG interface, the electropositive oxygen vacancies migrate from the top surface of the LAO toward the bottom 2DEG interface. Since the oxygen vacancies provide the hopping sites for electrons, the downward migration of the oxygen vacancies can be considered as the forming process of a conducting filament, that is the on-switching. On the other hand, when a positive voltage is applied, the oxygen vacancies move away from the interface, resulting in the off-switching. The asymmetry of the hysteresis is attributed to different band offsets at the top Pt/LAO and the bottom LAO/STO interfaces. Moreover, since the charge screening lengths are different at each interface, the effect of the applied electric field naturally depends on the direction of the field. Despite the asymmetry, we found that the conductivity of the 2DEG memristor can be switched by both positive and negative voltage pulses, revealing potential applications for analog memristors (Fig. S6, Supporting Information).

The gradual change of *I*_*v*_ at the high voltage regime in Fig. 2a gives us a hint that we can effectively implement multiple conductance states. Figure 2b shows the multiple conductance states of the same device, programmed by different writing voltage *V*_*write*_. We first fully turned off the device by applying the *V*_*write*_ of + 9 V and then gradually turned the device on by applying incremental *V*_*write*_. For each conductance state, *I*_*v*_ is measured at a reading voltage *V*_*read*_ of + 0.5 V. We could implement 27 discrete conductance states with the on/off ratio of 2.84 × 10^3^% at the *V*_*write*_ ranged from − 3.00 to − 4.25 V. In this conductance range, the switching characteristics are quite reliable and reproducible. The retention and the endurance properties are additionally described in Sect. 6 of Supporting Information. Notably, the overall change of the conductance value is desirably linear. In artificial neural networks, considering the required linear relationship between the input signals and the weight change for the network training, the linear dependence of conductance on *V*_*write*_ enables more efficient and accurate training (Sect. 7 in Supporting Information). Therefore, the high linearity of the conductance change, without additional doping^29^ or multilayer stacking^30^, makes this 2DEG memristor a promising candidate for synaptic applications. In fact, a larger number of conductance states (up to 52) were achieved in the same device with a broader range of *V*_*write*_. However, when the *V*_*write*_ increased over − 4.25 V, the conductance value was found to be not highly reproducible. Thus, for the following study, we consider only this linear and reliable conductance regime (the 27 conductance states).

Besides the remarkable resistive switching characteristics, it should be noted that the variance of the output current at each conductance state is not ideally small. The relatively large variance of output signal has been a nuisance not only for our device but for most of the newly proposed resistive switching devices as well^10,31,32^. We quantify the variance of the output signals from our device by calculating the averaged conductance value and the standard deviation at all individual states (Fig. 2c). The standard deviation clearly increases with the conductance of the device. The standard deviation values at the states 23–27 are comparable with the conductance difference between the neighboring states, indicating that the fluctuation during the read operation can occasionally degrade the precision of the 2DEG memristor. This large conductance variance is inevitable at the high-conductance regime (Sect. 8, Supporting Information). To further clarify the noise characteristics, we measured the current power spectral density (PSD) *S*_*I*_ (*f*) of the 2DEG memristor. Figure 2d shows the PSD spectra at 4 distinct conductance states. After setting each conductance state, the PSD spectrum of the *I*_*v*_ was measured at + 1 V. All the PSD spectra show a typical 1/*f* behavior, indicating the presence of charge traps with a wide range of time constants. It is also clearly seen that the fluctuation of *I*_*v*_ becomes stronger as the device conductance increases. Therefore, to fully take advantage of the resistive switching properties, we confront this noise and the resultant state-overlap issue.

### Variance-aware quantization

**Figure 3:**
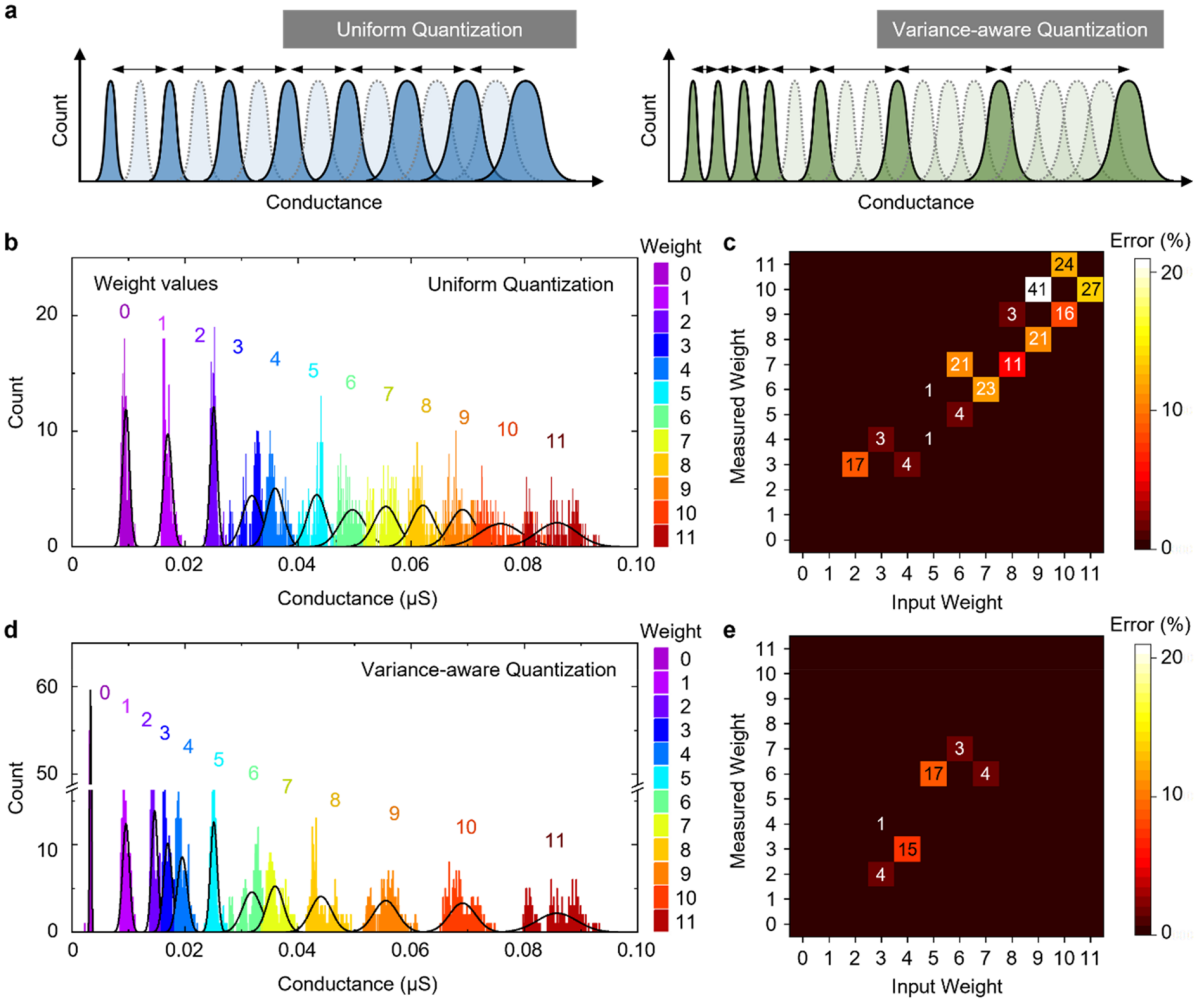
Variance-aware weight quantization for the 2DEG memristors. (**a**) Schematics showing the conventional uniform quantization and the variance-aware quantization methods. (**b**) Conductance histogram of uniformly-separated 12 conductance states. The black lines represent the normal distribution fitting curves. (**c**) Simulated heatmap of measurement error, calculated based on the uniformly-separated states. (**d**) Conductance histogram of the nonuniformly-separated 12 conductance states. The separation between each state is set small in the low current regime, while it is expanded as the current increases. (**e**) Simulated heatmap of measurement error, calculated based on the nonuniformly-separated states. Note that the error is significantly reduced by the variance-aware quantization method.

In principle, the state-overlap issue can be simply resolved if we selectively use only the conductance states whose distributions do not overlap with each other at all. Thus, in the case of previous memristors, the weight values are assigned to uniformly- and coarsely-defined conductance states. This conventional method limits the number of available states, degrading the fundamental performance of the memristors. Therefore, we propose the VAQ, a non-uniform quantization method designed to address the overlap issue. The non-uniform quantization has been used in different fields like image processing^33,34^, signal processing^35,36^, and deep learning^37,38^. Figure 3a schematically depicts the state configuration for the conventional uniform quantization and the VAQ. The conductance states are non-uniformly defined for the VAQ, such that the conductance distributions hardly overlap across any two neighboring states.

As a proof of concept, we select 12 non-uniform VAQ states out of the total 27 states (Fig. S10, Supporting Information) and then find the same number of uniformly-separated states (Fig. 3b,d) within the 27 states. We do not use the entire 27 conductance states but only a subset of them, so that we can define the same number of states in both the uniform and the non-uniform quantization schemes. Although these 12 states do not represent the full performance of our 2DEG memristors, they can directly reveal the impact of the VAQ as compared to the conventional uniform quantization within the given on/off ratio. To estimate the quantization errors depending on the state configuration, we measured 80 conductance values at each state and assigned them to the nearest conductance state. The heatmaps of the incorrectly quantized weight values when using the uniform states and the non-uniform states are given in Fig. 3c,e, respectively. The horizontal and the vertical axis of the heatmaps represent the intended weight values and the actually-quantized weight values, respectively. These heatmaps clearly show that the VAQ with the non-uniform states reduces the quantization errors effectively. Because the VAQ conductance states are configured to have minimal overlaps between the data distributions, the measured weight values are less likely assigned to the incorrect neighboring states, achieving lower quantization errors. The difference between the two quantization schemes is particularly significant at states 9–11, where the uniformly-separated states have largely overlapped distributions. This result empirically proves that the conductance states defined in a variance-aware manner exploit the available conductance range without suffering from the distribution overlap issue.

### Evaluation of the VAQ: matrix–matrix multiplication

**Figure 4:**
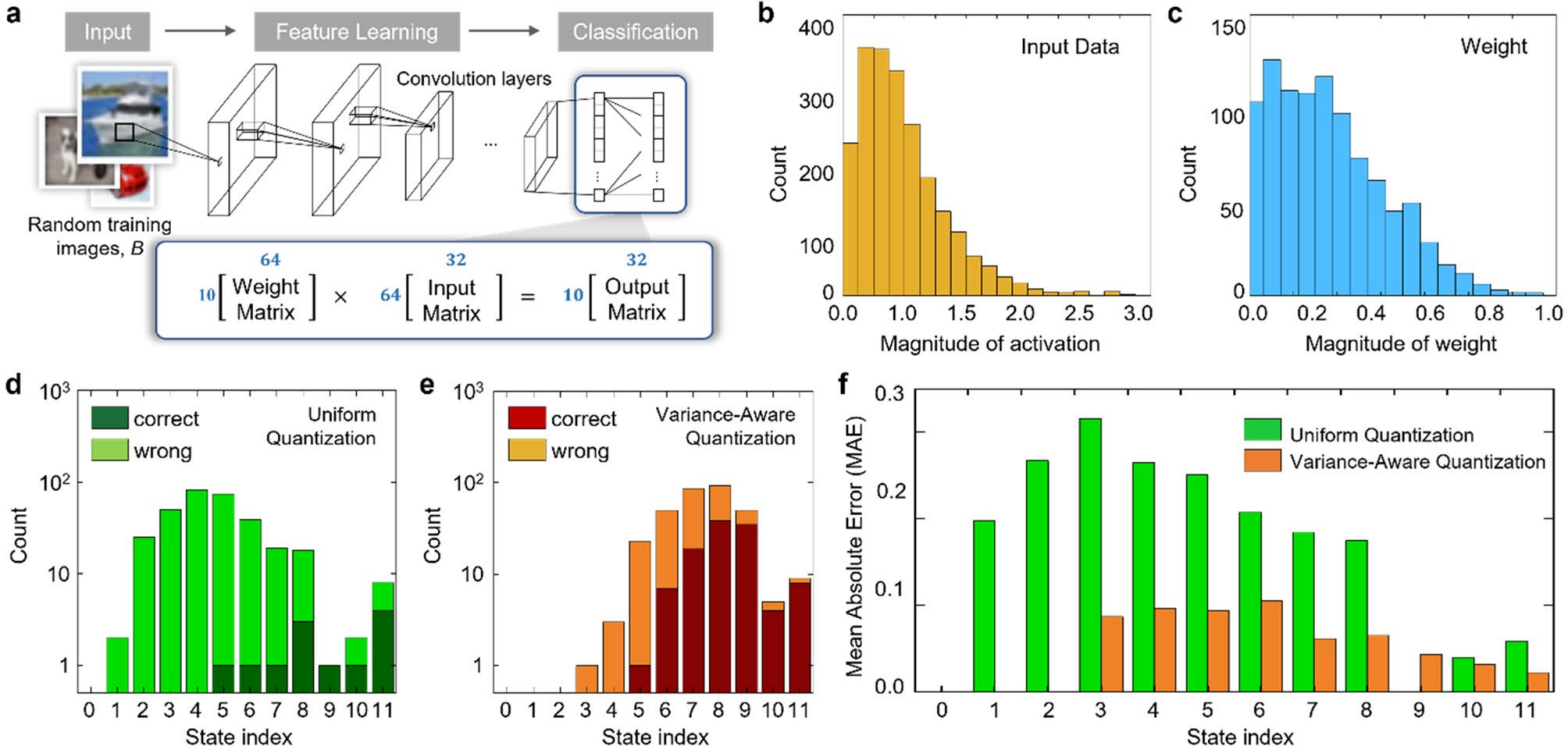
Variance-aware weight quantization for image classification problems. (**a**) An illustration of basic matrix operations for neural network training. The output activation matrix (10 × 32) is computed by multiplying the weight matrix (10 × 64) by the input activation matrix (64 × 32). We collected the data from a neural network designed for image classification tasks (ResNet20 output layer). (**b**) Histograms of the input data (i.e., activations from the previous layer) at the ResNet-20 output layer during training on CIFAR-10 dataset. (**c**) Histograms of the weight values at the same ResNet-20 output layer during training on CIFAR-10. (**d**) Histograms of the number of correct/wrong element-wise uniform quantization of the output matrix. We first get the ground-truth output matrix by quantizing the product of the two floating-point input matrices. We consider the quantization is correct if the quantized output element is the same as the corresponding ground-truth element. (**e**) Histograms of the number of correct/wrong element-wise variance-aware quantization of the output matrix. (**f**) Mean Absolute Error (MAE) of the output matrix. The error is calculated for 12 states separately.

To verify the advantage of the VAQ, we perform matrix–matrix multiplication using the conductance states of the 2DEG memristor defined by the uniform quantization and the VAQ. Figure 4a schematically shows how we extract the experimental data from a convolutional neural network (CNN)^39^. To demonstrate the VAQ performance under realistic settings, we use the data collected from the actual ResNet20 training on CIFAR-10 dataset (Sect. S10, Supporting Information)^40,41^. The histogram of the collected input activations and the weight values are given by Fig. 4b,c, respectively. They have a normal-like distribution as expected. We multiply these two matrices to obtain the output activation. To identify the impact of different quantization schemes, we calculate the output activations using the quantized input activation and weight values, and then compare them to the ground-truth. The output activation calculated using the non-quantized input activation and weight values is considered as the ground-truth.

Figure 4d,e show the number of the correctly- and wrongly-quantized elements of the output activations using the uniform quantization and the VAQ, respectively. Note that the uniformly-configured states do not appropriately represent the original data. Due to the strong overlap in the small-value regime, most of the quantized elements are assigned to the incorrect neighbor states. Even though this error rate is somewhat exaggerated due to the small number of available states (i.e., 12), it is clear that the quantization error is severe in this conventional quantization scheme. On the other hand, the VAQ method remarkably reduces the error. This comparison indicates that the VAQ can improve the quantization accuracy by resolving the state-overlap issue. The advantage of the VAQ can also be revealed by examining the mean absolute error (MAE). The MAE for each state, calculated using the different state configurations, are given in Fig. 4f. While the uniform quantization yields high errors especially in the small-value regime, the VAQ method markedly reduces the errors. The VAQ slightly increases the errors in the large-value regime because it has fewer states for it. Nonetheless, the overall errors are sufficiently small to be ignored, as compared to that in the uniform quantization method.

### Evaluation of the VAQ: a heavily re-used convolution filter

We point out another important advantage of the VAQ approach. Notably, the non-uniformly-configured conductance states for the VAQ can better represent the practical data distribution. In general, the weight values as well as the intermediate data, generated in neural network training, have normal-like distributions (Fig. S11, Supporting Information). The conventional uniformly-configured conductance states can well represent the large weight values, that take up only a small portion of the entire model parameters, while having significant quantization errors for the many small weight values (Fig. S12, Supporting Information). On the contrary, the non-uniform VAQ conductance states are suitable to represent such normal-like data distributions. Because a larger number of states is assigned to the small weight values than to the large weight values, such a non-uniform state configuration is advantageous for representing the normal-like data distribution. Therefore, the VAQ will allow the 2DEG memristors to achieve the higher classification and regression performances.

**Figure 5:**
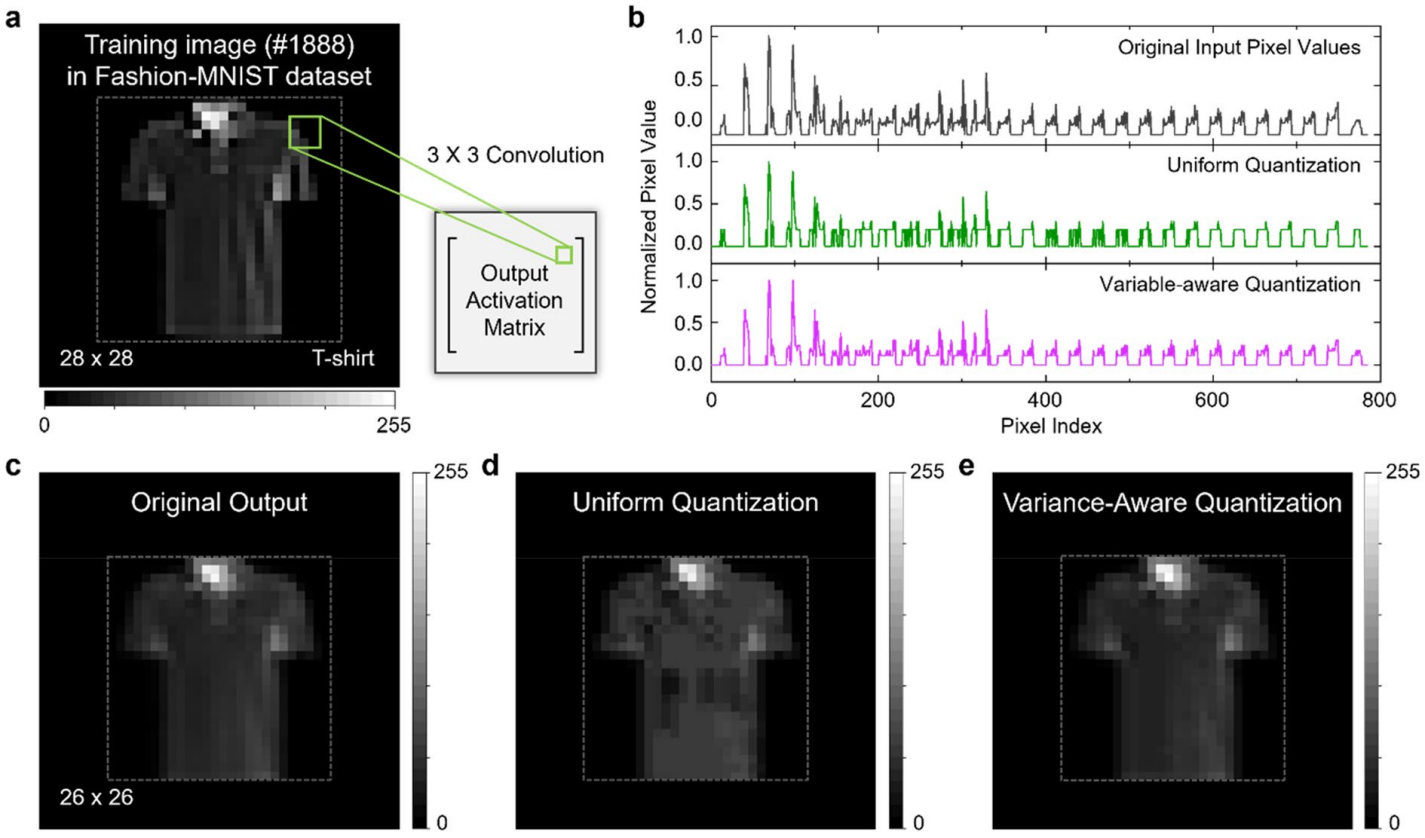
Variance-aware weight quantization for the convolution operation. (**a**) The training sample image (image #1888) of Fashion-MNIST dataset. The image consists of 28 × 28 gray-colored pixels. We apply a 3 × 3 convolution filter (stride of 1 × 1) to the input image and compare the output. (**b**) Normalized original input pixel values (top), the input pixel values quantized using the uniform states (middle), and the input pixel values quantized using the variance-aware states (bottom). (**c**) The original output of the convolution operations without quantization. (**d**) The output obtained by applying the uniform quantization. (**e**) The output obtained by applying the VAQ.

To directly demonstrate this advantage, we perform convolution operations using different state configurations for the uniform quantization and the VAQ. Figure 5a shows a training image, sample #1888 from Fashion-MNIST dataset^42^. The inset schematically shows the calculation of the output activation matrix from the input training image. The detailed simulation procedure is described in Sect. 13 of Supporting Information. The top panel of Fig. 5b shows the numerical input values for each pixel of the image. Note that most of the normalized data are lied between 0.2 and 0.6. This implies that the realistic data are not uniformly distributed over the entire range. To simulate the convolution operation of our 2DEG memristors, we quantized the non-uniformly distributed input data to 12 states before applying the convolution filter. The middle panel of Fig. 5b shows the quantized input data using the conventional uniform quantization method. It is clearly shown that the uniform quantization leads to loss of a significant amount of information in the input data. A majority of the input activations are quantized into smaller state values than their actual values due to the limited number of states for the small data values. On the other hand, the quantization error is effectively reduced by the VAQ. The bottom panel of Fig. 5b shows the quantized input data using the VAQ method. Unlike the uniform quantization, the finer-grained states for the small data values represent the input data more accurately. Particularly, while the uniform quantization zeroes out many small input data, the VAQ keeps them in non-zero states mitigating the loss of information.

As a reference, the original output data of the convolution operation without any quantization process is given in Fig. 5c. Since we apply a 3 × 3 convolution filter on the image with a stride of 1 × 1, the input image matrix of size 28 × 28 provides an output activation matrix of size 26 × 26. Figure 5d,e present the quantized convolution outputs computed based on the uniform quantization and the VAQ method, respectively. The uniform quantization fails to precisely represent the small output values making the overall image speckled and noisy, while the VAQ provides a comparable output image to the original output. These simulation results clearly show that the VAQ can effectively reduce the quantization errors in the convolution operation and, hence, is advantageous in terms of accuracy.

Note that we have not considered any specific activation function in this simulation. If an activation function was used, such as sigmoid or hyperbolic tangent, the output activations would be rescaled to a range of 0–1. That is, the data distribution can be shifted to the small value regime, and the uniform quantization likely loses more information compared to the VAQ. Likewise, if rectified linear unit (ReLU) was used^43^, the magnitude of the overall data flow would be significantly reduced since all the negative values are zeroed out. Regardless of the type of activation function, therefore, the VAQ is expected to result in smaller quantization errors than those of the uniform quantization.

## Conclusion

In conclusion, we demonstrate a novel memristive device based on the Pt/LAO/STO heterostructures. The voltage-driven migration of the surface oxygen vacancies enables controlling the tunneling of 2DEG in the Pt/LAO/STO junctions, achieving multiple non-volatile conductance states, over 27, with high reliability. The multi-level switching capability of the 2DEG memristor allowed us to explore an advanced weight quantization strategy, the VAQ. Our simulation studies verify that the VAQ can effectively reduce the state-overlap issue of the 2DEG memristors. In addition, since the VAQ states better represent the normal-like data distribution, the VAQ can significantly improve the computing accuracy of the 2DEG memristors. Although we demonstrated only the limited number of VAQ states for performance verification, we believe that the 2DEG memristors can implement a larger number of VAQ states and thereby achieve higher performance for practical applications. Lastly, we address that the VAQ states can be simply implemented by pre-defining the optimal writing voltages, without additional integration with other circuits. This implies that the VAQ method is readily applicable to other conventional resistive switching devices. Therefore, our results will provide a stepping stone for developing high-performance multi-bit memristors based on complex oxide heterostructures as well as other novel materials.

## Methods

### Device fabrication

LAO thin films were epitaxially grown on TiO_2_-terminated STO (001) substrates using PLD. To obtain the TiO_2_-terminated STO substrates, as-received STO substrates were etched using buffered-HF for 1 min and thermally annealed at 1000 °C for 6 h. To grow LAO films, a single-crystalline LAO target was ablated using a KrF (248 nm) excimer laser at a repetition rate of 3 Hz and with a fluence of 2.0 J/cm^2^. During the growth, the temperature of PLD chamber was kept as 550 °C. The oxygen partial pressure for growing LAO was 10^–3^ mbar. After growing the LAO films, the samples were slowly cooled down to room temperature (for ~ 2 h) without changing oxygen partial pressure. Subsequently, the 50-nm-thick Pt electrodes were fabricated by a conventional lift-off process.

### Scanning transmission electron microscopy (STEM) measurements

A cross-section specimen for TEM analysis was fabricated by using a focused ion beam (FIB) machine (Helios G4, FEI), and the sample thickness of sub 30 nm was achieved via FIB milling. The LAO/STO interface structure was measured at the atomic resolution using a Titan Double Cs corrected TEM (Titan cubed G2 60–300, FEI) in high-angle annular dark field scanning TEM (HAADF-STEM) mode. The microscope was operated at 200 kV accelerating voltage with the beam convergence semi-angle of 17.9 mrad. The inner and outer angles of the HADDF detector were chosen as 40 and 200 mrad, respectively. A 2048 × 2048 image of the LAO/STO interface was measured with 1 µs dwell time and 8.1 pm pixel size. The total electron dose was about 1.91 × 10^5^ electrons Å^−2^.

### X-ray diffraction (XRD) measurements

The lattice structure of the LAO thin film was determined by XRD. A D8 Discover (Bruker AXS) high-resolution X-ray diffractometer with a Cu K_α_ source (λ = 1.5405 Å) was used for the XRD measurements. The out-of-plane *θ*–2*θ* XRD pattern of the as-grown LAO/STO heterostructure was measured at around (002) STO peak.

### Electrical characterizations

The *I–V* measurements and the multi-level switching experiments were conducted using a semiconductor analyzer, Keithley 4200. For all the electrical characterizations, an input voltage (i.e.,* V*_*write*_ or *V*_*read*_) is directly applied to the bottom 2DEG interface and the output current is measured through the top Pt contact. To minimize the contact resistance, we widely deposited a commercial silver paste onto the side wall of the sample and contacted the silver surface using a commercial metal prove tip. As for the *I–V* curve measurements, the input voltage was swept from − 9 to + 9 V and vice versa. Totally, 50 cycles of *I–V* curves are successively measured without changing the environment. To demonstrate the capability of multi-level switching, we initialized the 2DEG memristor by applying + 9 V to the bottom contact. By this initialization process, the device was completely turned off. Subsequently, we gradually switched the device on by applying an incremental voltage from − 3 to − 4.25 V. The conductance states were identified by measuring the *V*_*read*_ of + 0.5 V.

### Noise characterizations

The electrical noise spectra of the device were measured using a dynamic signal analyzer, SR785 (Stanford Research Systems) along with a low-noise current amplifier, SR 570 (Stanford Research Systems). After programming the conductance state by applying different *V*_*write*_, the current power spectral density was measured at a constant voltage of + 1 V. The maximum frequency was set as 102.4 kHz.

## Supplementary Information

Supplementary Information.

## Data Availability

The data that support the findings of this study are available from the corresponding authors upon request.

## References

[1] Chua L (1971). Memristor—The missing circuit element. IEEE Trans. Circuit Theory.

[2] Strukov DB, Snider GS, Stewart DR, Williams RS (2008). The missing memristor found. Nature.

[3] Prezioso M (2015). Training and operation of an integrated neuromorphic network based on metal-oxide memristors. Nature.

[4] Yao P (2020). Fully hardware-implemented memristor convolutional neural network. Nature.

[5] Ielmini D, Wong HSP (2018). In-memory computing with resistive switching devices. Nat. Electron..

[6] Li C (2018). Analogue signal and image processing with large memristor crossbars. Nat. Electron..

[7] Lee MJ (2011). A fast, high-endurance and scalable non-volatile memory device made from asymmetric Ta_2_O_5__−__x_/TaO_2__−__x_ bilayer structures. Nat. Mater..

[8] Sheridan PM, Cai F, Du C, Ma W, Zhang Z, Lu WD (2017). Sparse coding with memristor networks. Nat. Nanotechnol..

[9] Chanthbouala A (2012). A ferroelectric memristor. Nat. Mater..

[10] Schranghamer TF, Oberoi A, Das S (2020). Graphene memristive synapses for high precision neuromorphic computing. Nat. Commun..

[11] Zhu X, Li D, Liang X, Lu WD (2019). Ionic modulation and ionic coupling effects in MoS2 devices for neuromorphic computing. Nat. Mater..

[12] Terabe K, Hasegawa T, Nakayama T, Aono M (2005). Quantized conductance atomic switch. Nature.

[13] Xue W (2020). Controllable and stable quantized conductance states in a Pt/HfOx/ITO memristor. Adv. Electron. Mater..

[14] Yi W (2016). Quantized conductance coincides with state instability and excess noise in tantalum oxide memristors. Nat. Commun..

[15] Yu S (2012). Characterization of low-frequency noise in the resistive switching of transition metal oxide HfO 2. Phys. Rev. B.

[16] Jacob, B. *et al*. Quantization and training of neural networks for efficient integer-arithmetic-only inference. *Proceedings of the IEEE Conference on Computer Vision and Pattern Recognition* (2018).

[17] Franchi, G., Bursuc, A., Aldea, E., Dubuisson, S. & Bloch, I. TRADI: Tracking deep neural network weight distributions. *Computer Vision–ECCV 2020: 16th European Conference, Glasgow, UK, August 23–28, 2020, Proceedings, Part XVII 16* (Springer International Publishing, 2020).

[18] Bellido, I. & Fiesler, E. Do backpropagation trained neural networks have normal weight distributions? *International Conference on Artificial Neural Networks* (Springer, 1993).

[19] Nakagawa N, Hwang HY, Muller DA (2006). Why some interfaces cannot be sharp. Nat. Mater..

[20] Lee H (2018). Direct observation of a two-dimensional hole gas at oxide interfaces. Nat. Mater..

[21] Ohtomo A, Hwang HY (2004). A high-mobility electron gas at the LaAlO_3_/SrTiO_3_ heterointerface. Nature.

[22] Brinkman A (2007). Magnetic effects at the interface between non-magnetic oxides. Nat. Mater..

[23] Reyren N (2007). Superconducting interfaces between insulating oxides. Science.

[24] Mannhart J, Schlom DG (2010). Oxide interfaces—An opportunity for electronics. Science.

[25] Cheng G (2011). Sketched oxide single-electron transistor. Nat. Nanotechnol..

[26] Wu S (2013). Nonvolatile resistive switching in Pt/LaAlO_3_/SrTiO_3_ heterostructures. Phys. Rev. X.

[27] Mitra C, Lin C, Robertson J, Demkov AA (2012). Electronic structure of oxygen vacancies in SrTiO_3_ and LaAlO_3_. Phys. Rev. B.

[28] Zhong Z, Xu PX, Kelly PJ (2010). Polarity-induced oxygen vacancies at LaAlO_3_/SrTiO_3_ interfaces. Phys. Rev. B.

[29] Chandrasekaran S, Simanjuntak FM, Saminathan R, Panda D, Tseng TY (2019). Improving linearity by introducing Al in HfO_2_ as a memristor synapse device. Nanotechnology.

[30] Jiang Y (2021). Linearity improvement of HfOx-based memristor with multilayer structure. Mater. Sci. Semicond. Process..

[31] McConville JP (2020). Ferroelectric domain wall memristor. Adv. Funct. Mater..

[32] Yeon H (2020). Alloying conducting channels for reliable neuromorphic computing. Nat. Nanotechnol..

[33] Mehmood A, Khan IR, Dawood H, Dawood H (2021). A non-uniform quantization scheme for visualization of CT images. Math. Biosci. Eng..

[34] Cai, J. & Zhang, L. Deep image compression with iterative non-uniform quantization. In* 2018 25th IEEE International Conference on Image Processing *(*ICIP*) 451–455 (2018).

[35] Khan S, Goodall RM, Dixon R (2013). Non-uniform sampling strategies for digital control. Int. J. Syst. Sci..

[36] Beyrouthy, T., Fesquet, L. & Rolland, R. Data sampling and processing: Uniform vs. non-uniform schemes. In *2015 International Conference on Event-based Control, Communication, and Signal Processing *(*EBCCSP*) 1–6 (2015).

[37] Li, Y., Dong, X. & Wang, W. Additive powers-of-two quantization: An efficient non-uniform discretization for neural networks. In *International Conference on Learning Representations* (2019).

[38] Baskin C (2021). Uniq: Uniform noise injection for non-uniform quantization of neural networks. ACM Trans. Comput. Syst. (TOCS).

[39] LeCun Y (1989). Backpropagation applied to handwritten zip code recognition. Neural Comput..

[40] He, K., Zhang, X., Ren, S. & Sun, J. Deep residual learning for image recognition. In *Proceedings of the IEEE Conference on Computer Vision and Pattern Recognition* (2016).

[41] Krizhevsky, A. & Hinton, G. Learning multiple layers of features from tiny images 7 (2009).

[42] Xiao, H., Rasul, K. & Vollgraf, R. Fashion-mnist: A novel image dataset for benchmarking machine learning algorithms. arXiv:1708.07747 (2017).

[43] Nair, V. & Hinton, G. E. Rectified linear units improve restricted Boltzmann machines. *Icml* (2010).

