# Supplementary material for: Variance-aware weight quantization of multi-level resistive switching devices based on Pt/LaAlO3/SrTiO3 heterostructures
Source: Sci Rep. 2022 May 31;12:9068. doi: 10.1038/s41598-022-13121-4 (PMC9156742; doi:10.1038/s41598-022-13121-4)
Supplement: Supplementary file 1 — Supplementary Information. [file 41598_2022_13121_MOESM1_ESM.docx]

**Supporting Information**

**Variance-aware Weight Quantization of Multi-level Resistive Switching Devices based on Pt/LaAlO_3_/SrTiO_3_ Heterostructures**

Sunwoo Lee^1^, Jaeyoung Jeon^2,3^, Kitae Eom^4^, Chaehwa Jeong^5^, Yongsoo Yang^5^, Ji-Yong Park^2,3^, Chang-Beom Eom^4^, and Hyungwoo Lee^2,3^*

^1^ Department of Electrical and Computer Engineering, University of Southern California, Los Angeles, CA 90007, USA

^2^ Department of Physics, Ajou University, Suwon, 16499, Republic of Korea

^3^ Department of Energy Systems Research, Ajou University, Suwon, 16499, Republic of Korea

^4^ Department of Materials Science and Engineering, University of Wisconsin-Madison, Madison, WI 53706, USA

^5^ Department of Physics, Korea Advanced Institute of Science and Technology (KAIST), Daejeon, 34141, Republic of Korea

* E-mail: hyungwoo@ajou.ac.kr

**1. Resistive switching mechanism of the 2DEG memristor**


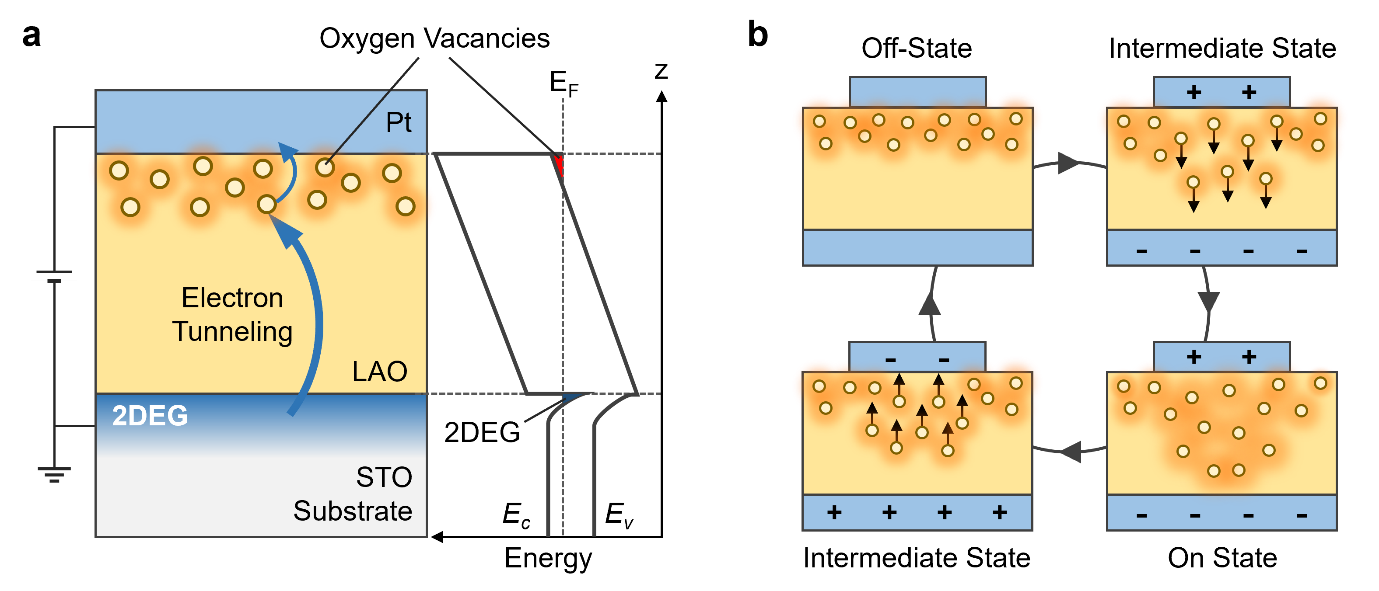


**Figure S1.** **(a)** Schematic diagram showing the tunneling device configuration of a Pt/LAO/STO heterostructure. The inset shows the energy band diagram of the Pt/LAO/STO heterostructure. **(b)** The conductance switching mechanism based on the migration of oxygen vacancies in the LAO.

Figure S1(a) shows the schematics depicting the tunneling device configuration of the Pt/LAO/STO heterostructure. When a positive bias voltage is applied to the top Pt electrode, or a negative bias voltage is applied to the bottom 2DEG interface, the electrons from the 2DEG interface tunnel through the LAO layer, resulting in a tunneling current. We employ oxygen vacancy point defects to modulate the tunneling (*i.e.,* vertical) current. Because the oxygen vacancies form intermediate energy levels within the LAO bandgap, the electron tunneling probability can be tuned by redistributing the oxygen vacancies in the LAO layer.

Figure S1(b) shows the hypothetical mechanism for the resistive switching in the Pt/LAO/STO heterostructure. The switching mechanism is expected to be similar to that for conventional resistive switching devices based on TiO_2_ or Ta_2_O_5_. However, the unique and interesting difference of the LAO/STO heterostructure is that the as-grown LAO thin film readily has oxygen vacancies at the top surface. This is because the oxygen vacancy formation energy is lowest at the top surface of the LAO due to the internal built-in electric field (see the band diagram of the Figure S1(a)). According to the well-known polar catastrophe mechanism, the surface oxygen vacancies can be considered as a counterpart of the 2DEG at the bottom interface. In the as-grown LAO/STO heterostructure, the surface oxygen vacancies are far away from the bottom interface and, hence, do not significantly contribute to the tunneling conduction. Therefore, the initial state of the device is the off-state. If we apply a high negative voltage to the bottom 2DEG interface, the electropositive oxygen vacancies migrate towards the bottom interface. Because the distance between the oxygen vacancies, working as electron hopping sites, and the 2DEG interface becomes shorter, the tunneling probability and tunneling current will increase. Thus, applying a pulse of high negative voltage can be considered as a switching-on process. Depending on the magnitude of the voltage, the effective distance between the electron hopping sites and the bottom 2DEG interface will be changed. This allows us to implement multiple conductance states.

We observed that when the writing voltage is over a certain value (*V_write_* = -4.25 V in our case), the output current became unstable and noisy. Thus, we define the conductance state written by the *V_write_* of -4.25 V as the complete-on state. This switching mechanism may look similar to the well-known resistive switching mechanism based on a conducting filament. However, we believe that there are a few different aspects in our oxide heterostructures. For example, since we utilize the surface oxygen vacancies and do not induce additional redox reaction, the switching characteristics are highly reproducible. In addition, we do not perform the well-known forming process to initiate the conducting filament in the insulating oxide layer. By the forming process, while the on-state current can be secured, the noise issue is unavoidable. When the filament is disconnected from one side of the junction, the incomplete conducting paths cause the strong electrical noise, resulting in the low reliability. However, in our devices, we do not form the fully-connected filament across the Pt/LAO/STO junction. By controlling the oxygen-deficient region only near the top surface of the LAO, we precisely tune the tunneling of the 2DEG through the ultrathin LAO barrier.

**2. Complementary heterointerfaces in Pt/LAO/STO heterostructures**


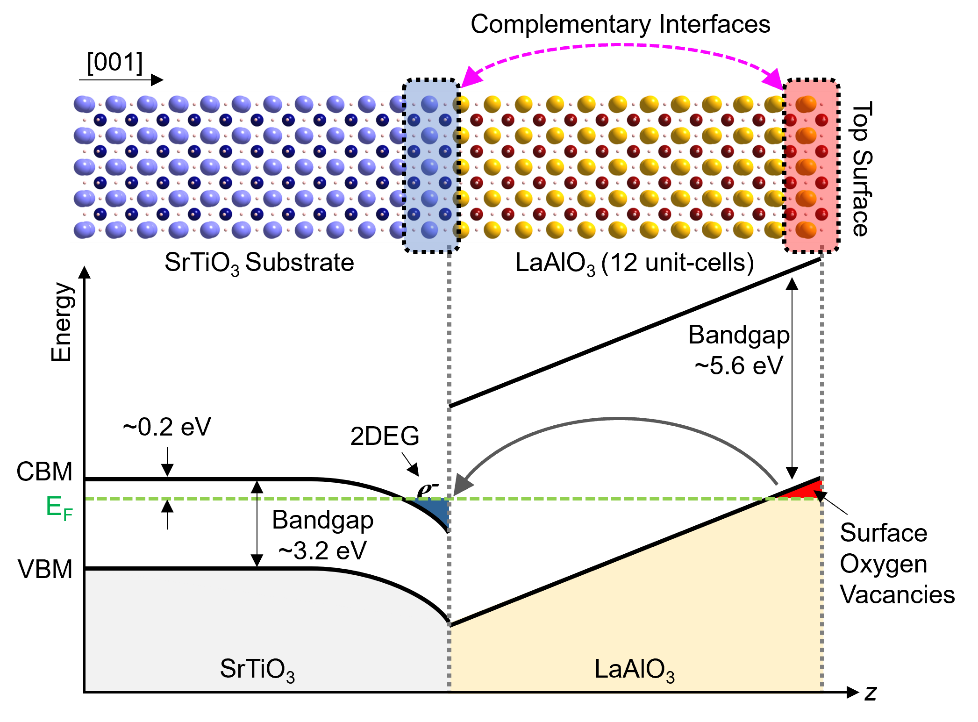


**Figure S2.** Schematic energy band diagram of the LAO/STO heterostructure.

The emergence of the 2DEG at oxide interfaces is often explained by the ‘polar catastrophe’ mechanism. In this model, when a positively charged atomic layer (*i.e.,* [LaO]^+^ of LaAlO_3_) forms an interface with a charge-neutral atomic layer (*i.e.,* [TiO_2_]^0^ of SrTiO_3_), an electric field that points away from the interface to the top surface is generated due to the polarity discontinuity. This internal field is compensated at the interface by the electron accumulation, that is the 2DEG (Figure S2). As a counterpart, electropositive oxygen vacancies are spontaneously formed at the top surface (the red triangular region). This is a quite strong constraint. Recent studies confirm that the 2DEG concentration of ~0.5 electron/unit-cell (~3.4 x 10^14^ cm^-2^) is indeed correlated with the oxygen vacancy concentration of ~0.25 vacancy/unit-cell (~1.7 x 10^14^ cm^-2^) in LAO/STO heterostructures.^[44]^ Note that the internal electric field in the LAO/STO heterostructures directly depends on the LAO thickness. Therefore, we can reproducibly tune the amount of surface oxygen vacancies by controlling the LAO thickness.

**3. The effect of the bottom Ag contact**


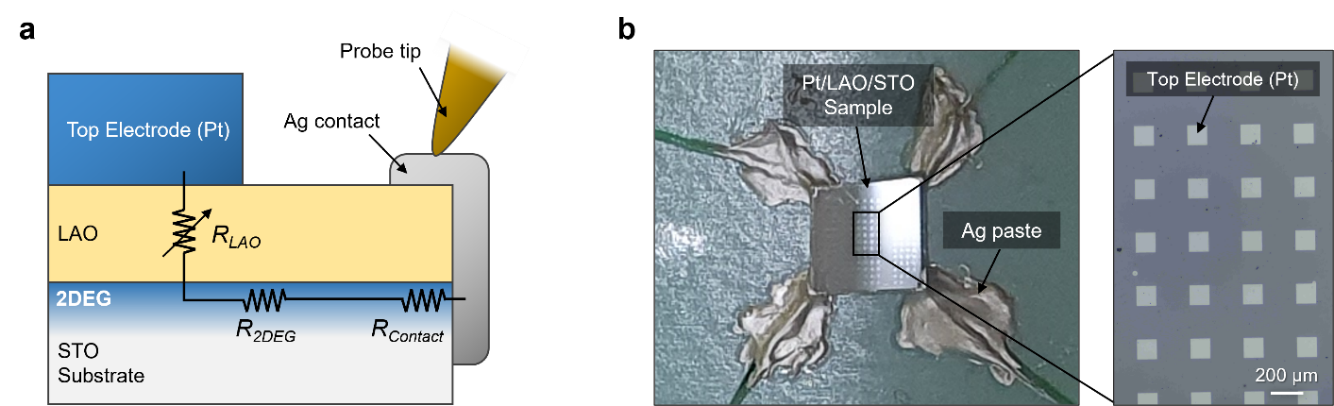


**Figure S3. (a)** Schematic illustration of the electrical contact configuration. The *R_LAO_* represents the switchable resistance of the device. The *R_2DEG_* and *R_Contact_* are the resistance of the 2DEG at the interface and the contact resistance between the 2DEG and the Ag, respectively. Both resistances are small enough to be ignored in this device. **(b)** Optical images of the electrical contacts on the Pt/LAO/STO heterostructure.

Figure S3 shows the detailed contact structure of the 2DEG memristors. The square-patterned Pt pads and the conducting 2DEG at the LAO/STO interface work as the top and the bottom electrodes, respectively. To make an ohmic contact to the 2DEG, we used a commercial Ag paste. To clarify the effect of the metal contacts, we prepared another Pt/LAO/STO heterostructure and fabricated Au contacts using a wire-bonder. Figure S4a and S4b show the *I-V* characteristics of the 2DEG memristors with the original Ag and the new Au contacts, respectively. The pinched hysteresis loops and the bipolar resistance switching behaviors are almost identical to each other, indicating that the original Ag contacts did not influence the resistive-switching mechanism.


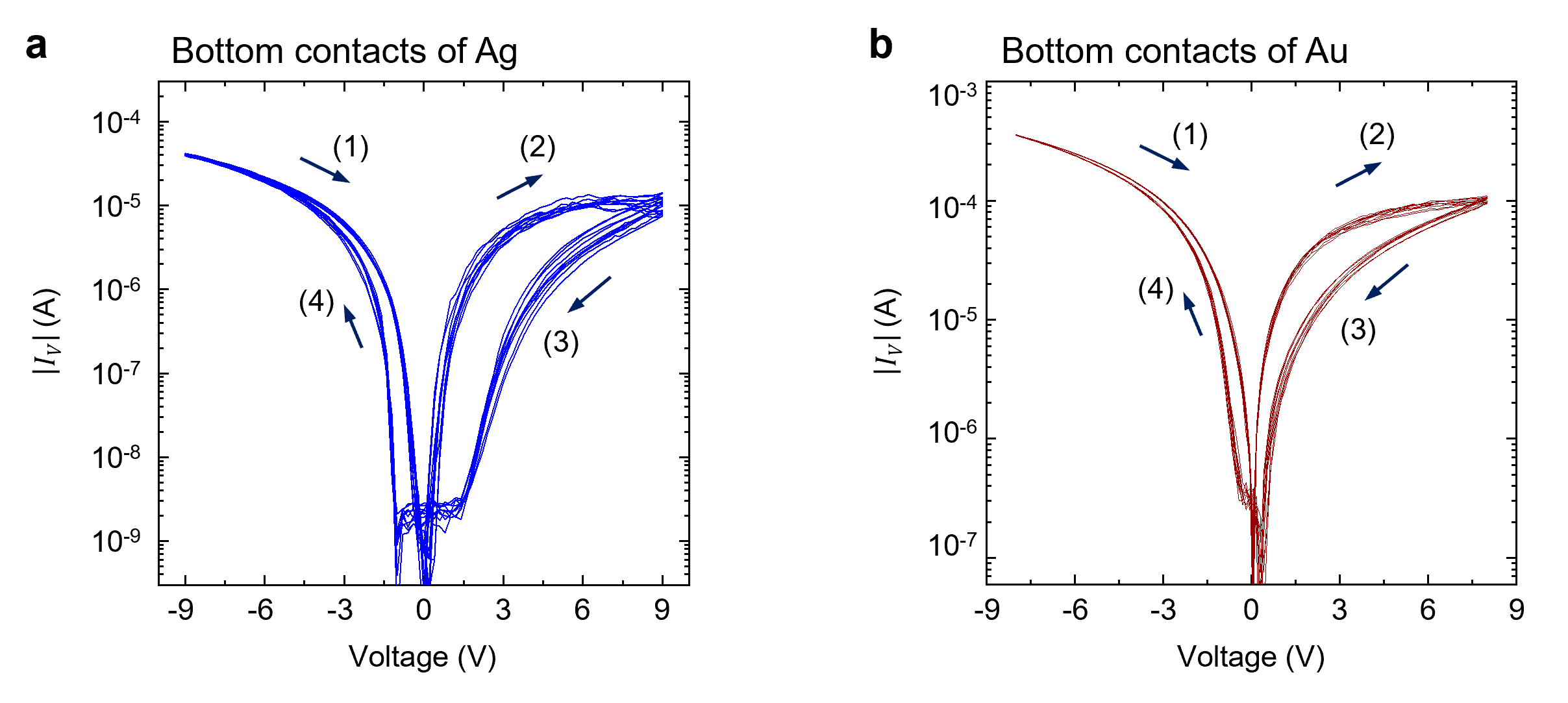


**Figure S4 (a)** The *I-V* curves of the 2DEG memristor with Ag contacts. The *I-V* curves were measured 10 times consecutively. **(b)** The *I-V* curves of the 2DEG memristor with Au contacts. Note that the asymmetric hysteresis behaviors are almost identical with each other, indicating that the Ag ions do not involve the resistive-switching mechanism.

**4. Initial conductance state of the Pt/LAO/STO heterostructures**


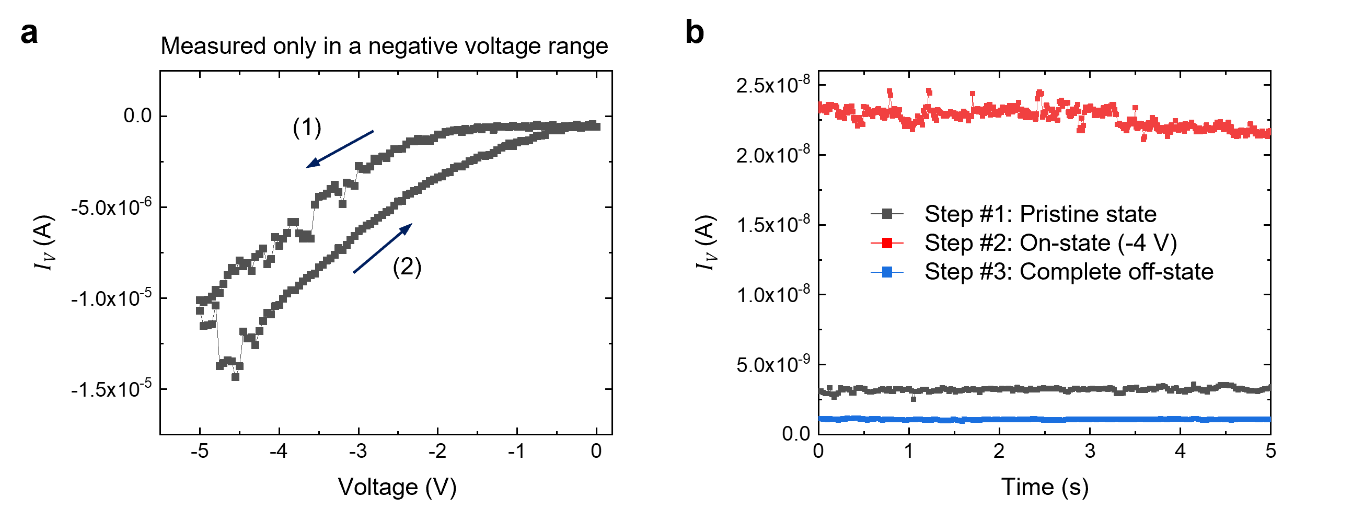


**Figure S5 (a)** The *I-V* curve measured from a pristine Pt/LAO/STO heterostructure. To clarify the initial conductance state, we swept the input voltage within the negative voltage range only. **(b)** Current traces measured from another pristine sample. Note that the conductance at the pristine state is similar to that at the complete off-state. The *V_read_* is fixed at 0.5 V.

Figure S5a shows the initial *I-V* characteristics of a pristine Pt/LAO/STO heterostructure. To clarify the initial conductance state of the device, we swept the input voltage from 0 V to -5 V and back to 0 V. The counterclockwise hysteresis shows that the conductance of the device was initially low and increased by the negative voltage sweep. Figure S5b shows the current traces measured from another pristine sample. The current at a fixed voltage of 0.5 V was sequentially measured at the pristine state (gray), after the on-switching process (red), and after the off-switching process (blue). Note that the initial conductance of the device was quite low and similar to that at the complete off-state. The small conductance difference between the initial state and the complete off-state is presumably due to the relatively broader distribution of the surface oxygen vacancies in the pristine sample. These results consistently show that the initial conductance state of the device is the off-state, which support our switching mechanism based on the surface oxygen vacancies.

**5. Analogue switching characteristics of the 2DEG memristors**


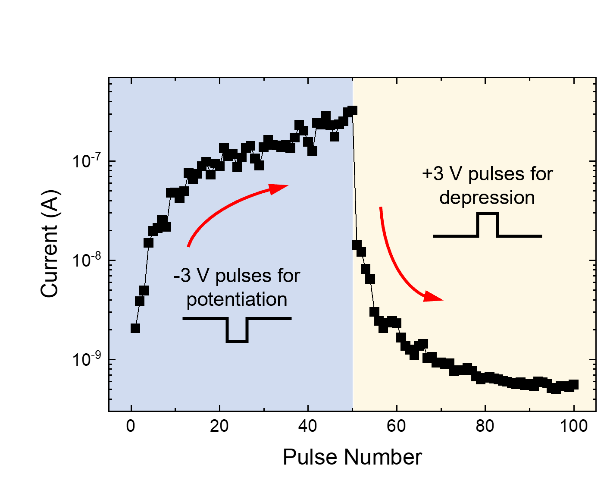


**Figure S6.** Analog conductance update characteristics of the 2DEG memristor. We consecutively applied 50 pulses of -3 V (300 ms) and 50 pulses of +3 V (800 ms) for the analogue switching test. The output current was measured after each pulse with the reading voltage of 0.5 V. The device showed the gradual conductance update at both switching polarities, revealing potential applications for analog memristors.

**6. The retention and the endurance properties of the 2DEG memristors**


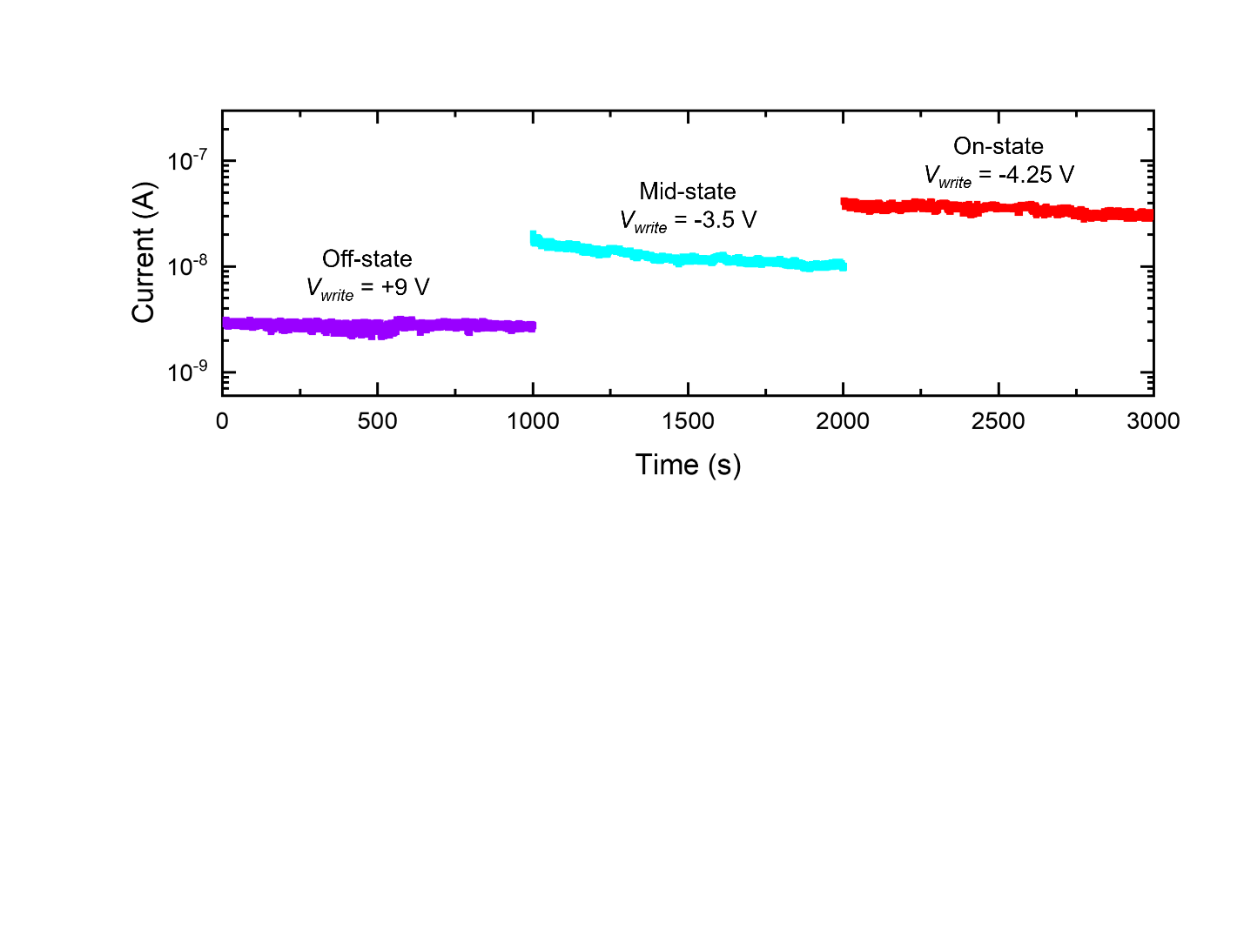


**Figure S7.** The retention property of the 2DEG memristor. After switching the device to each conductance state, the output current was measured at *V_read_* = 0.5 V for 1000 s.

We performed the retention test using the 2DEG memristor. After switching the device to the representative 3 states (the off-, the mid- and the on-states), the output current was measured at *V_read_* = 0.5 V for over 1000 sec (Figure S7). We confirmed that the retention of those states is at least 10^3^ s. The change in conductance with time is sufficiently small as compared to the conductance window between each state.

We also performed the repetitive switching test using the same device. Figure S8 shows the consecutively measured 70 switching cycles. The off- and the on-states were programmed by voltage pulses of *V_write_* = +9 V (150 ms) and -4.25 V (270 ms), respectively. The output current was measured after each writing voltage pulse with a reading voltage pulse of 0.5 V (150 ms). Notably, the device showed a minimal degradation during the 70 switching cycles.


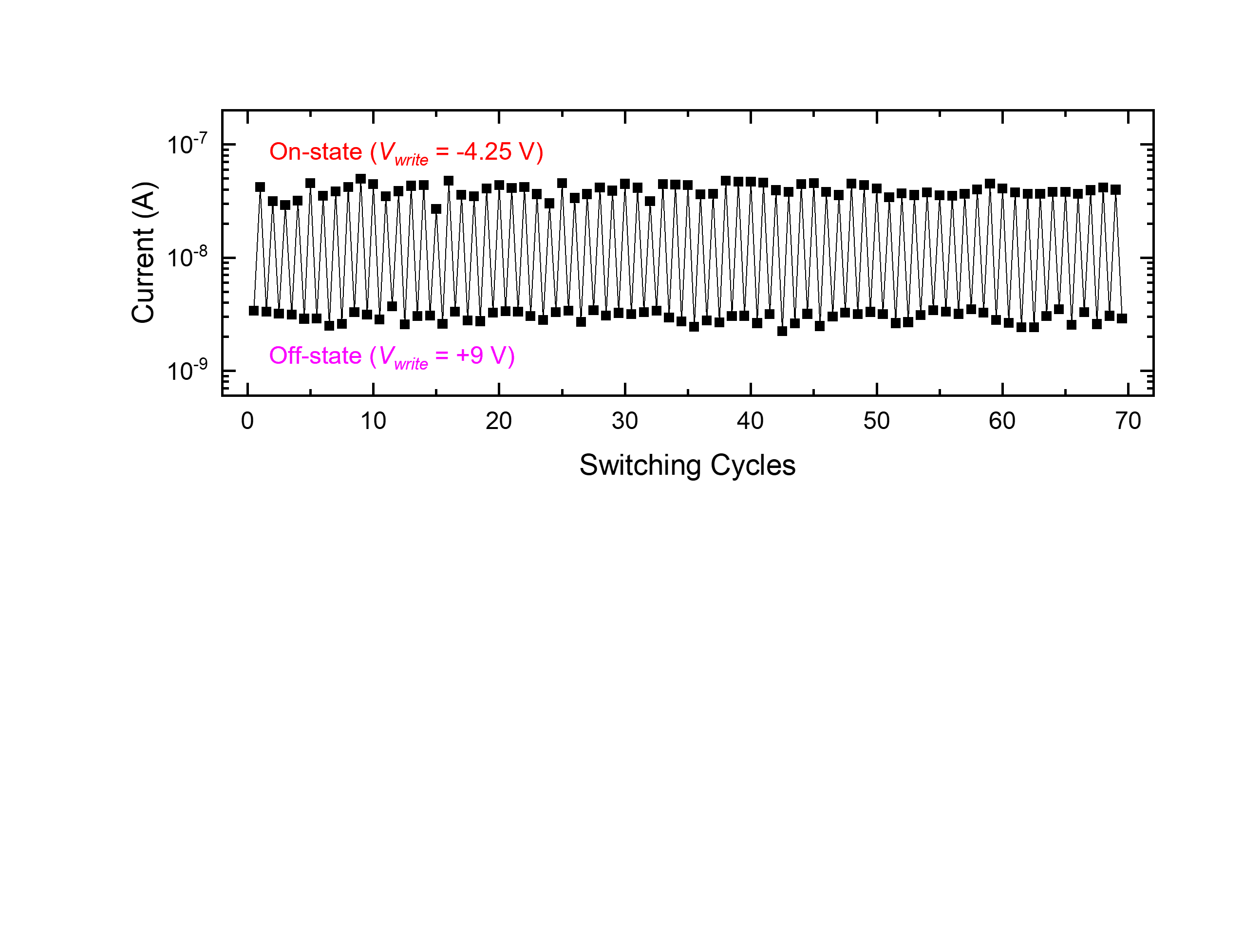


**Figure S8.** The endurance test of the same device. Note that the device showed a minimal degradation during the 70 switching cycles.

**7. Stochastic gradient descent (SGD)-based neural network training**

Linearity between activations and weight change is essential for accurate neural network training. When training neural networks using SGD, one of the standard training algorithms, the weight change is first-order gradient of the weight, which is linear to the activations. Therefore, the linear conductance change of memristors enables to precisely implement the gradient-based training algorithm.

The gradients of the objective function with respect to the weight parameters are calculated as follows.

$$\frac{\partial C}{\partial w^{l}}=\frac{\partial C}{\partial a^{l}}\frac{\partial a^{l}}{\partial w^{l}}, l\in\left\{ 1, \ldots,L \right\},$$

where *L* is the number of layers, $C=\frac{1}{2}\left( a^{L}-\hat{a}^{L} \right)^{2}$ is the objective function, $w^{l}$is the weight parameters at layer $l$, and $a^{l}$is the activations at layer $l$. Note that $\frac{\partial C}{\partial a^{L}}=a^{L}-\hat{a}^{L}$, which is usually called ‘error’, is calculated at the output layer and backpropagated through all the layers. Because $a^{l}=w^{l}a^{l-1}+b^{l}$, $\frac{\partial a^{l}}{\partial w^{l}}=a^{l-1}$. Consequently, the above gradient can be re-written as follows.

$$\frac{\partial C}{\partial w^{l}}=\delta^{l}a^{l-1}, l\in\left\{ 1,\ldots,L \right\},$$

where $\delta^{l}$ is the error at layer $l$. The above equation shows that the gradient is linear to the activations propagated from the previous layer and the errors backpropagated from the next layer. Finally, the parameter update rule of SGD is as follows.

$$w_{k}^{l}=w_{k-1}^{l}-\eta\frac{\partial C}{\partial w_{k-1}^{l}},$$

where $k$ is the training iteration index and $\eta$ is the learning rate. This update rule clearly shows why the linearity between the weight change (gradient) and the activations is desired to implement accurate neural network training. The weight values are adjusted using the scaled gradient, and thus the weight change is always linear to the activations. The linear and gradual conductance change capability of our memristor enables to precisely implement this parameter updates.

**8. Noise characteristics of the 2DEG memristor**

As shown by Figure 2c, the current fluctuation becomes larger as the device conductance increases. This is consistent with what we observed from the current power spectral density (current PSD: $S_{I}(f)$) measured from the same device (Figure 2d). The current spectra showed a typical 1/*f* noise feature, implying that there exists a large number of charge traps around the device channel. When the charge carrier number is $\Delta N$ and the electrical current is *I*, the current PSD generated by the device can be written as $S_{I}\left( f,x,y,z \right)=I^{2}\frac{S_{N_{t}}(f,x,y,z)}{{(\Delta N)}^{2}}$, where the $S_{N_{t}}$is the PSD of mean-square fluctuation in the number of occupied charge traps in the device. According to the Wiener–Khinchin theorem, the $S_{N_{t}}$ can be written as

$$S_{N_{t}}\left( f,x,y,z \right)=\int\int\frac{4\tau(E,x,y,z)}{1+{[2\pi f\cdot\tau(E,x,y,z)]}^{2}}\times f_{t}(1-f_{t})N_{t}(E,x,y,z)dEdV$$

The *N_t_*, $\tau$, *f*, $f_{t}$, and *V* are the density of charge traps over the space and energy, a trapping time constant, a frequency, the trap occupancy function, and the effective volume of the device channel, respectively. Assuming that the number of charge traps and the total number of charges do not change, the current fluctuation is proportional to the square of the device current. This means that the noise issue at the high-conductance regime is inevitable and hinders us to use those conductance states. We believe that this issue is not only for our device, but for all of the similar resistive-switching devices in general.


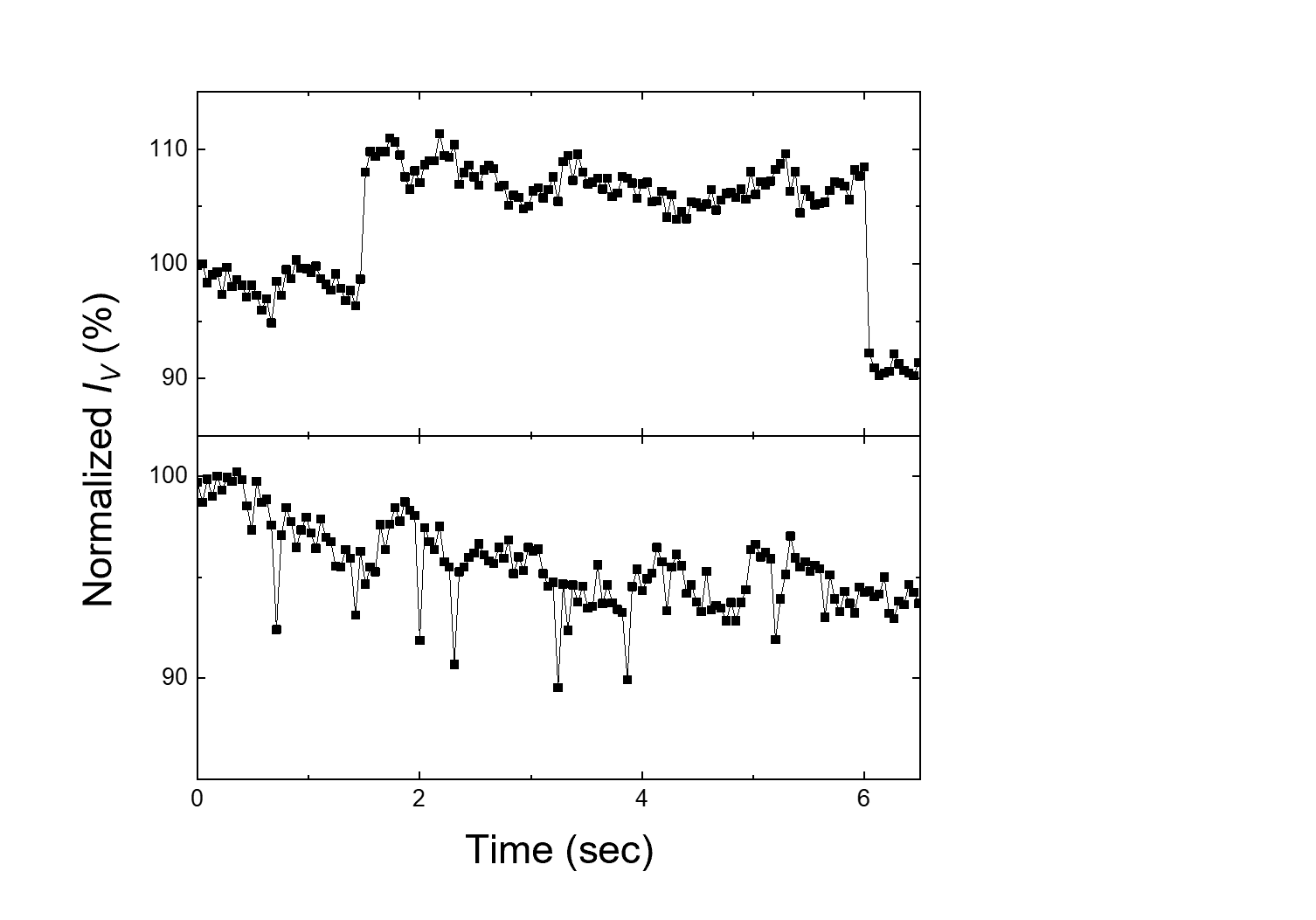


**Figure S9.** The RTS-like noise observed in the output signal. To better represent the noise characteristics the output current is normalized.

In addition, it should be also noted that we have observed the random telegraph signal (RTS)-like noise in the output signal, as shown by Figure S9. This RTS-like noise became severer as the conductance state gets closer to the complete on-state, which implies that there are a number of imperfect conducting paths around the major conducting path. This issue also needs to be resolved. However, considering that most of the resistive switching devices use a large number of point defects or mobile ions to switch their resistance, it might be not so simple to satisfactorily resolve the issue. Therefore, it is important to find a method to circumvent the noise issues. Note that our VAQ approach provides an efficient way to alleviate the noise issues.

**9. The multiple conductance states of the 2DEG memristor**


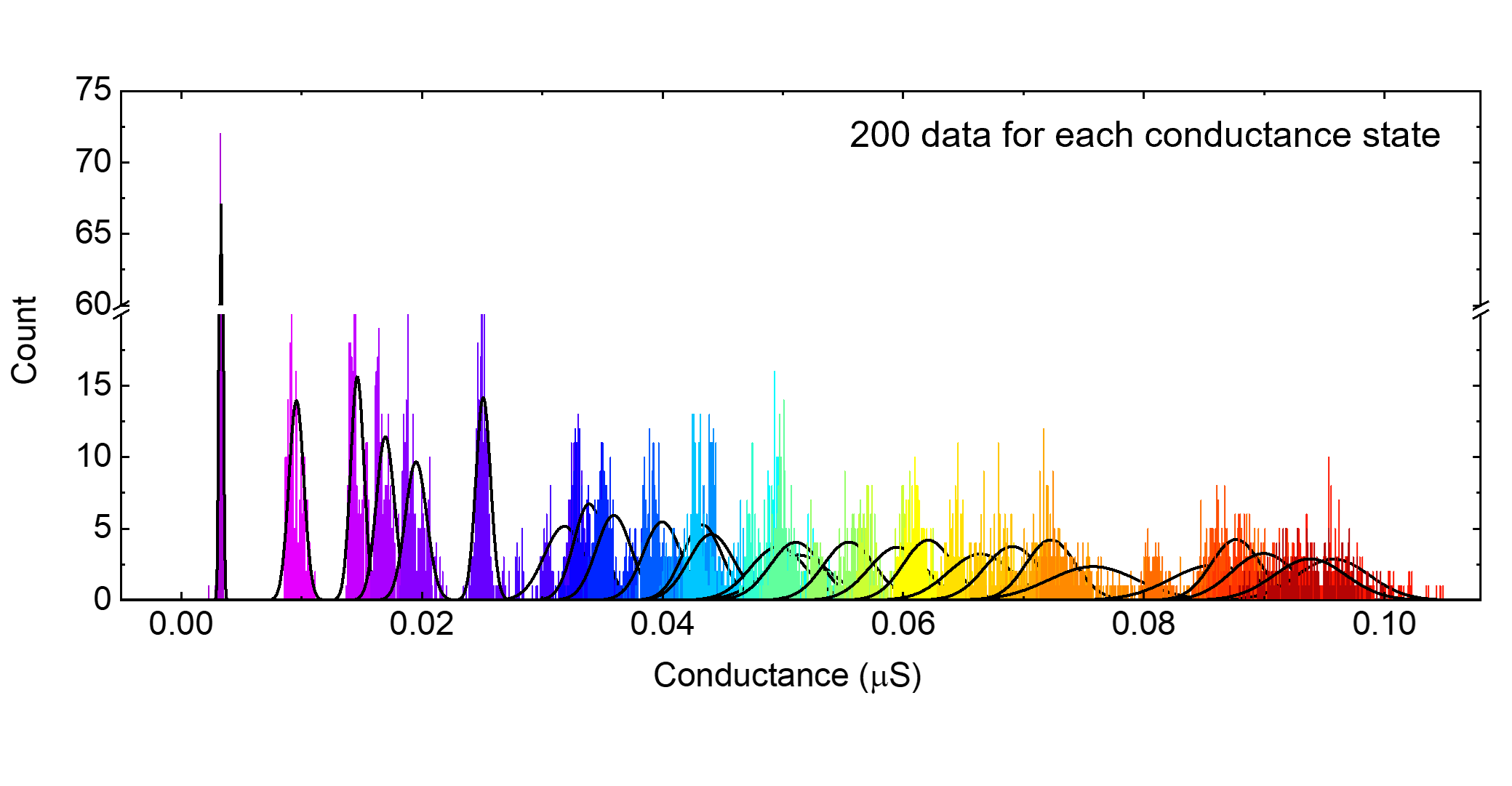


**Figure S10.** Histogram of the device conductance. Totally 27 discrete conductance states are represented. The complete off-state is achieved by applying +9 V to the top electrode of the 2DEG memristor. The remaining 26 on-states are achieved by applying *V_write_*, ranged from -3 V to -4.25 V, to the bottom electrode. The *V_read_* is 0.5 V. We acquired 200 conductance values for each state. Note that the overlap between the conductance states becomes severer as the device conductance increases.

**10. Matrix-matrix multiplication experiment**

The activations and weight values shown in Figure 3b and 3c are collected from ResNet20 model training. We implement ResNet20 training code using TensorFlow^[45]^ 2.4.0. The activations and the weights are collected from the last fully-connected layer of the model after training on CIFAR-10 dataset for 10 epochs. The model is trained using mini-batch stochastic gradient descent with momentum. The mini-batch size, the learning rate, and the momentum coefficient are set to 128, 0.1, and 0.9, respectively. The model parameters are initialized using Glorot uniform distribution.^[46]^ Each mini-batch is randomly extracted from the given 50,000 training images. Once the activations and the weights are collected, we first replace all the negative data with its absolute values. Then, we normalize the data such that all the collected data lie between 0 and 1. This normalization step makes the whole data distribution covered by the quantization states without losing any information.

To evaluate the performance of the quantization schemes, we prepare three sets of the output matrices. The first one is the ground-truth output matrix that is a product of the floating-point input activation matrix and the weight matrix. We call this original output matrix ***G*** for convenience. The second one is a product of the two input matrices quantized using the uniform quantization method. We call the output matrix ***U***. The third one is a product of the two input matrices quantized using the VAQ method. We call this output matrix ***V***. Once the three output activation matrices are obtained, we first quantize ***G*** and ***U*** using the uniform quantization. Then, compare each element between the two quantized output matrices. We consider the element is correctly quantized if it is fallen into the same state for both matrices. We count the correctly quantized elements for each state (Figure 3d). Likewise, we also quantize ***G*** and ***V*** using the VAQ and then count the correctly quantized elements for each state (Figure 4e).

Finally, we calculate the state-wise mean absolute error (MAE) using the three quantized output matrices (Figure 4f). The state-wise MAE between ***G*** and ***U*** is calculated using the following equation.

$e_{i}=\frac{1}{N_{i}}\sum_{j=1}^{N_{i}} \left| g_{i}^{j}-u_{i}^{j} \right|$,

where $N_{i}$ is the number of elements in ***G*** quantized into $i^{th}$ state, $g_{i}^{j}$ is the $j^{th}$ element in ***G*** quantized to $i^{th}$ state, and $u_{i}^{j}$ is the corresponding $j^{th}$ element in ***U***. The MAE between ***G*** and ***V*** is obtained in the same way.

**11. Data distribution of weight parameters**


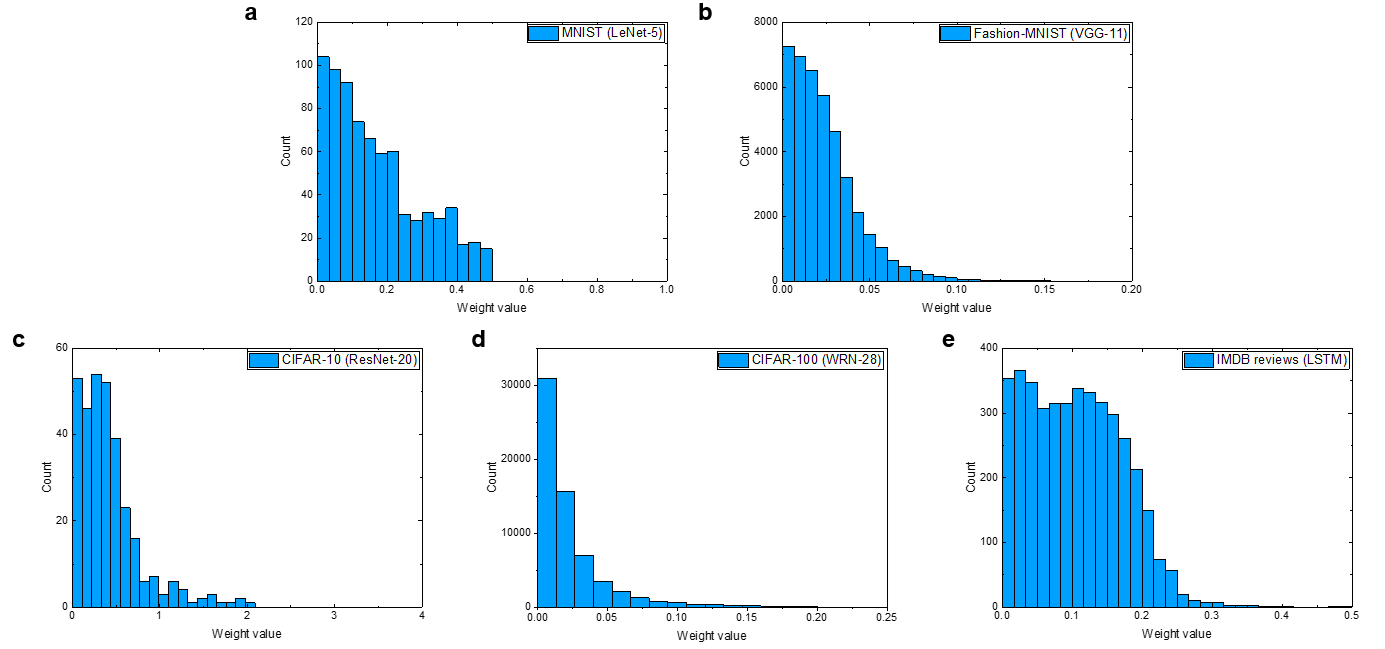


**Figure S11.** The weight values collected from various neural networks. **(a)** LeNet-5 training on MNIST. **(b)** VGG-11 training on Fashion-MNIST. **(c)** ResNet20 training on CIFAR-10. **(d)** WideResNet28-10 training on CIFAR-100. **(e)** LSTM training on IMDB. All the weights are collected from the largest fully-connected layer in the model.

We present the general data distribution of the weight parameters using popularly used benchmark datasets and networks. Figure S11 present five histograms of the output layer weight parameters collected from LeNet-5,^[47]^ VGG-11,^[48]^ ResNet20, WideResNet-28-10, and LSTM.^[49]^ These five networks are trained on MNIST,^[47]^ Fashion-MNIST, CIFAR-10, CIFAR-100,^[41]^ and IMDB review,^[50]^ respectively. We collect the weight values after 10 epochs of training from all the five networks.

Regardless of the model architecture and the dataset, the weight parameters have a normal-like distribution. Since we do not consider the negative values, the distribution has a one-sided bell shape. This observation motivates us to design the non-uniform quantization method in a variance-aware manner. Such normal-like distributions can be better represented by the non-uniform conductance states that cover the small-value regime more finely than the large-value regime.

**12. Impact of quantization methods on non-uniform data distribution**


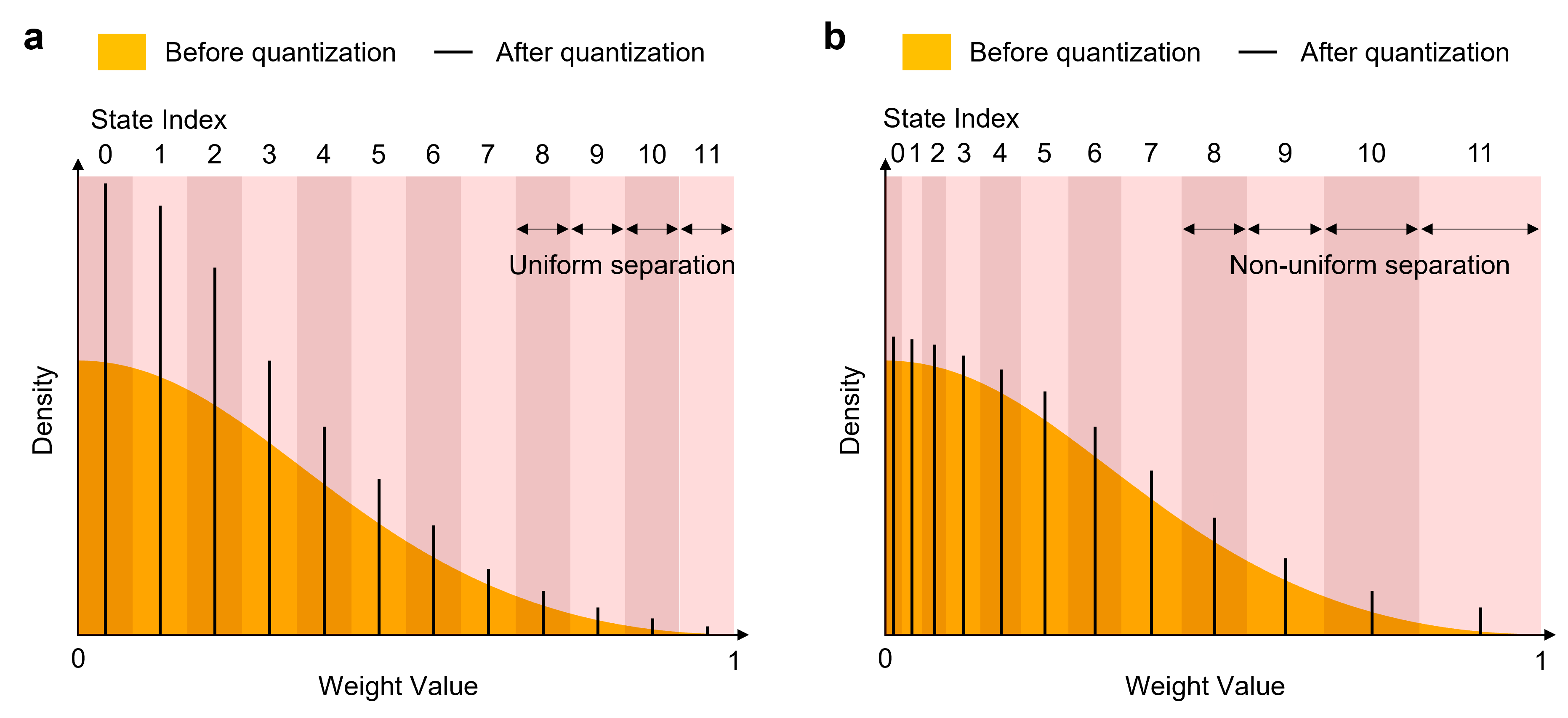


**Figure S12.** **(a)** Uniform and **(b)** non-uniform weight quantization with 12 states. When the available conductance range is uniformly separated to have the 12 states, many small weight values have a large quantization error. In contrast, when the conductance range is non-uniformly separated, the small values are well represented while slightly increasing the quantization errors for the large weight values. Since the data has a normal-like distribution, the non-uniform quantization provides a smaller total quantization error than the uniform quantization.

Figure S12a and S12b show example illustrations of the uniform quantization and the VAQ. We define 12 discrete states to quantize the distributed data (the orange-colored), ranging from 0 to 1. In the case of the conventional uniform quantization, the 12 states are defined with uniform separation. On the other hand, the 12 states are unevenly defined for the VAQ, such that the weight difference between two consecutive states increases as the state index increases. The black bars represent the density of the quantized data for each state. While the same normal-like data distribution is given, the uniform-quantization states poorly represent the original data distribution while the non-uniform quantization states more precisely represent the original data. Unless the data distribution is uniform, the appropriately defined non-uniform states are supposed to achieve smaller quantization errors than the uniform-quantization. It has been empirically found that the data distribution of weight parameters in neural networks have a normal-like distribution as shown in Figure S12. That is, the uniformly distributed conductance states are sub-optimal for memristors.

**13. Convolution experiment**

Figure 5a depicts the computations performed at the first convolution layer of a convolutional neural network. In our simulation, we first chose an arbitrary training image (#1888 training sample) from Fashion-MNIST dataset as the input data. Given the original floating-point input pixel values, we applied the uniform quantization and the VAQ to get the quantized input data (Figure 5b). Then, we applied a $3\times3$ convolution filter to the three input matrices using a stride of $1\times1$. The convolution filter was initialized using Glorot uniform distribution. Finally, each output matrix was converted back to a gray-color image to visualize the result. We use python OpenCV package^[51]^ to convert the output activation matrices to the image files.

**References**

44. Song, K. et al. Electronic and structural transitions of LaAlO_3_/SrTiO_3_ heterostructure driven by polar field‐assisted oxygen vacancy formation at the surface. *Adv. Sci.* **8**, 2002073 (2021).

45. Abadi, M. et al. Tensorflow: Large-scale machine learning on heterogeneous distributed systems. arXiv preprint arXiv 1603.04467 (2016).

46. Glorot, X., Bengio, Y. Understanding the difficulty of training deep feedforward neural, *Proceedings of the Thirteenth International Conference on Artificial Intelligence and Statistics* **9**, 249-256 (2010).

47. LeCun, Y., Bottou, L., Bengio, Y., & Haffner, P. Gradient-based learning applied to document recognition. *Proceedings of the IEEE* **86**, 2278-2324 (1998).

48. Simonyan, K., Zisserman, A. Very deep convolutional networks for large-scale image recognition. *arXiv preprint arXiv* 1409.1556 (2014).

49. Hochreiter, S., Schmidhuber, J. Long short-term memory. *Neural computation* **9**, 1735-1780 (1997).

50. Maas, A. L. et al. Learning word vectors for sentiment analysis. *Proceedings of the 49th annual meeting of the association for computational linguistics: Human language technologies* (2011).

51. Bradski, G. The openCV library. *Dr. Dobb's Journal: Software Tools for the Professional Programmer* **25**, 120-123 (2000).
